# T-cadherin modulates adipogenic differentiation in mesenchymal stem cells: insights into ligand interactions

**DOI:** 10.3389/fcell.2024.1446363

**Published:** 2024-12-09

**Authors:** Veronika Sysoeva, Ekaterina Semina, Polina Klimovich, Konstantin Kulebyakin, Valentina Dzreyan, Ekaterina Sotskaya, Anna Shchipova, Vladimir Popov, Alena Shilova, Ilya Brodsky, Nikita Khabibullin, Nikita Voloshin, Vsevolod Tkachuk, Kseniya Rubina

**Affiliations:** ^1^ Faculty of Medicine, Lomonosov Moscow State University, Moscow, Russia; ^2^ Institute of Medicine and Life Science, Immanuel Kant Baltic Federal University, Kaliningrad, Russia; ^3^ Chumakov Federal Scientific Center for Research and Development of Immune-and-Biological Products of Russian Academy of Sciences, Moscow, Russia

**Keywords:** adipogenesis, adipogenic differentiation, T-cadherin, adiponectin, low density lipoproteins, neural network quantification

## Abstract

**Introduction:**

T-cadherin, a non-canonical member of the cadherin superfamily, was initially identified for its involvement in homophilic recognition within the nervous and vascular systems. Apart from its adhesive function, T-cadherin acts as a receptor for two ligands: LDL, contributing to atherogenic processes, and HMW adiponectin, a hormone with well-known cardiovascular protective properties. However, the precise role of T-cadherin in adipose tissue remains elusive. Previously, we generated Cdh13^∆Exon3^ mice lacking exon 3 in the Cdh13 gene, which encodes the T-cadherin protein, and characterized their phenotype.

**Methods:**

Using wild-type (WT) and T-cadherin-deficient mice (Cdh13ΔExon3), we isolated and cultured mesenchymal stem cells to explore the role of T-cadherin in adipogenic differentiation. The experimental approaches employed include culturing cells under standard or adipogenic conditions, performing Oil Red O and Nile Red staining followed by quantitative analysis, conducting rescue experiments to reintroduce T-cadherin using lentiviral constructs in T-cadherin-deficient cells combined with automated adipocyte differentiation quantification via a neural network. Additionally, Western blotting, ELISA assays, and statistical analysis were utilized to verify the results.

**Results:**

In this study, we demonstrate for the first time that T-cadherin influences the adipogenic differentiation of MSCs. The presence of T-cadherin dictates distinct morphological characteristics in MSCs. Lack of T-cadherin leads to spontaneous differentiation into adipocytes with the formation of large lipid droplets. T-cadherin-deficient cells (T−/− MSCs) exhibit an enhanced adipogenic potential upon induction with differentiating factors. Western Blot, ELISA assays, and rescue experiments collectively corroborate the conclusion that T−/− MSCs are predisposed toward adipogenic differentiation. We carried out an original comparative analysis to explore the effects of T-cadherin ligands on lipid droplet accumulation. LDL stimulate adipogenic differentiation, while T-cadherin expression mitigates the impact of LDL on lipid droplet accumulation. We also examined the effects of both low molecular weight (LMW) and high molecular weight (HMW) adiponectin on lipid droplet accumulation relative to T-cadherin. LMW adiponectin suppressed lipid droplet accumulation independently of T-cadherin, while the absence of T-cadherin enhanced susceptibility to the suppressive effects of HMW adiponectin on adipogenesis.

**Discussion:**

These findings shed light on the role of T-cadherin in adipogenic differentiation and suggest an interplay with other receptors, such as LDLR and AdipoRs, wherein downstream signaling may be modulated through lateral interactions with T-cadherin.

## 1 Introduction

Adipose tissue, a subtype of connective tissue, encompasses a diverse array of cell types, including stem/progenitor cells, preadipocytes, mature adipocytes, fibroblasts, vascular endothelial cells, and immune cells (macrophages and leukocytes) (Merrick et al., 2019; Bäckdahl et al., 2021; Wang and Wang, 2023). Mature adipocytes accumulate lipid droplets, which function as the body’s primary energy reservoir. Throughout ontogenesis, adipose tissue exhibits a remarkable degree of plasticity, undergoing significant changes in metabolism, structure, phenotype, and secretome in response to various physiological stimuli (Sakers et al., 2022). In addition to its role as an energy reservoir through triglyceride accumulation, adipose tissue emerges as an important endocrine organ that regulates the overall body metabolism. Mature adipocytes produce a wide range of biologically active molecules referred to as adipokines (adiponectin, leptin, resistin, vaspin, etc.), growth and differentiation factors (FGF21, BMP4, etc.), and microRNAs (Guerre-Millo, 2004; Kershaw and Flier, 2004; Cao, 2014; Kurylowicz, 2021).

In normal metabolism, the physiological expansion of adipose tissue occurs through an increase in the number of adipocytes, a process known as hyperplasia. However, under chronic malnutrition leading to obesity, the increase in adipose tissue volume primarily occurs through hypertrophy, characterized by an enlargement of the preexisting adipocytes (Sakers et al., 2022). In cases of metabolic syndrome, obesity, and insulin resistance, alterations extend beyond mere changes in adipose tissue morphology. Deficiencies emerge in the renewal of adipose tissue cells, proliferation of stem/progenitor cells, differentiation and maturation of adipocytes. This also entails a disturbance in the hormonal and secretory functions of adipose tissue, resulting in changes in the spectrum of secreted adipokines, growth factors, and microRNAs (Kahn et al., 2019).

While adipose tissue may appear uniform at first glance, it is, in fact, remarkably heterogeneous in its cellular composition. Diverse identities of heterogeneous stem/progenitor cell subpopulations, characterized by the expression of specific markers and distinct differentiation trajectories, and committed cells have been discovered (Burl et al., 2018; Schwalie et al., 2018; Merrick et al., 2019; Khazaei et al., 2022). Additionally, multiple genetic lineage tracing and transcriptomics analyses revealed heterogeneity among mature adipocytes within the same depots, which, despite their similar size, exhibit distinct adipokine secretion profiles (Bäckdahl et al., 2021; Corvera, 2021).

Among adipokines produced by adipose tissue, adiponectin notably stands out due to its high concentration in blood, typically ranging from 0.01% to 0.05% and exceeding that of the other hormones and cytokines by 3–6 orders of magnitude (Maeda et al., 2020). Adiponectin plays systemic protective roles against diabetes and atherosclerosis by exerting insulin-sensitizing, anti-inflammatory, and anti-proliferative effects. Moreover, adiponectin enhances fatty acid oxidation in skeletal muscles, regulates blood sugar levels and reduces triglyceride accumulation (Mattu and Randeva, 2013; Khoramipour et al., 2021). Concentration of adiponectin is markedly reduced in pathological conditions, such as type 2 diabetes mellitus (T2DM), obesity, and cardiovascular diseases (Achari and Jain, 2017; Rubina et al., 2021). Adiponectin circulates in three distinct forms in the bloodstream, each possessing distinct biological properties: low molecular weight (LMW), trimer-dimer or middle molecular weight hexamer, and high molecular weight (HMW) form (Khoramipour et al., 2021). Numerous studies indicate that the HMW form of adiponectin exhibits the highest metabolic activity (Hara et al., 2006; Mattu and Randeva, 2013). Adiponectin has three major receptors, AdipoR1, AdipoR2, and T-cadherin (Khoramipour et al., 2021; Rubina et al., 2021). However, T-cadherin was shown to act as an exclusive receptor for hexametric and high-molecular-weight (HMW) adiponectin (Hug et al., 2004; Fukuda et al., 2017). It serves as a major binding partner for native adiponectin in serum, as demonstrated by specific interactions between native adiponectin and T-cadherin (Kita et al., 2019). Moreover, the same authors revealed that T-cadherin knockdown, rather than AdipoRs, significantly reduces the binding of native adiponectin to C2C12 myotubes, HEK293 and CHO cells (Kita et al., 2019). Additionally, single nucleotide polymorphism (SNPs) in *CDH13* gene encoding T-cadherin strongly correlates with plasma adiponectin level and cardiovascular diseases in humans (Rubina et al., 2021).

While adiponectin is predominantly synthesized by adipose tissue in adult organisms, the expression and function of its key receptor, T-cadherin, in this tissue remain elusive. Several single-cell RNA sequencing analyses reported T-cadherin expression in mesenchymal stem cells (MSCs) from various sources, indicating that MSCs express T-cadherin at least at the mRNA level. Specifically, T-cadherin mRNA has been detected in MSC cell clusters positive for PDGFRα (Pdgfrα) and Meflin (Islr) alongside well-known mesenchymal markers, such as CD73, CD90, and CD105 (Holley et al., 2015; Nakamura et al., 2020; Ramirez et al., 2020).

One of the most intriguing aspects of T-cadherin biology is its role as a receptor for two distinct ligands: HMW adiponectin and low-density lipoproteins (LDL) (Tkachuk et al., 1998; Rubina et al., 2021). Elevated levels of LDL in blood have been strongly linked to the pathophysiology of metabolic disorders, cardiovascular diseases, type 2 diabetes (T2D), insulin resistance (IR), and systemic inflammation (Kannel, 1987; Bissonnette et al., 2023). When contemplating the functional significance of T-cadherin as a dual receptor, we proposed a concept placing T-cadherin as a master switch. This concept implied that the interaction of T-cadherin with LDL or HMW adiponectin might determine, whether the outcomes are protective or detrimental, depending on the concentrations of these ligands in bloodstream (Balatskaya et al., 2019; Sysoeva et al., 2023).

To explore the potential involvement of T-cadherin in adipogenic differentiation, we performed a series of experiments utilizing MSCs isolated from the adipose tissue of both control and T-cadherin deficient mice. We examined the role of T-cadherin in adipogenic differentiation and lipid accumulation as well as the effects of T-cadherin ligands (LDL and adiponectin) on adipogenic differentiation. This approach aimed to gain deeper insights into whether T-cadherin orchestrates a feedback loop on adipogenesis upon binding with its ligands and to shed light on the potential function of T-cadherin in the adipose tissue.

## 2 Materials and methods

### 2.1 Animals

T-cadherin-deficient mice (Cdh13^DExon3^ mice) were generated as previously described by crossing Cdh13^loxP/loxP mice^, possessing 2 loxP sites flanking exon 3 of the Cdh13 gene, and mice constitutively expressing Cre-recombinase (Popov et al., 2023). Adult Cdh13^DExon3^ mice and wild-type (WT) C57BL/6J mice were maintained in standard polypropylene cages under controlled vivarium conditions (temperature: 20°C–24°C, humidity: 35%–65%, 12-h light/dark cycle) with *ad libitum* access to food and water. Animal care and handling adhered to the European Convention for the Protection of Vertebrate Animals used for Experimental and other Scientific Purposes (ETS №123). All procedures were complied with Directive 2010/63/EU of the European Parliament and the Council of 22 September 2010 on the protection of animals used for scientific purposes. Animal manipulations received ethical approval from the local ethical committee in accordance with the in-house requirements of the Commission on Bioethics of Lomonosov Moscow State University (license number 3.4).

### 2.2 Isolation and culturing of mesenchymal stem cells (MSCs)

Male 8-week-old mice were euthanized by injection of a lethal dose of Avertin. Subcutaneous adipose tissue from the thigh region was excised and placed in HBSS buffer (Gibco) containing 500 units/mL Antibiotic-Antimycotic (Gibco). The adipose tissue was mechanically minced to obtain a homogeneous mass and enzymatically disaggregated in HBSS buffer (Gibco) with addition of 200 units/mL type I collagenase (Worthington) and 30 units/mL neutral protease (Worthington) at 37°C for 1 h upon constant stirring. Cells were pelleted by centrifugation at 200 *g* for 10 min and cultured in control media (standard media composition: DMEM, 10% FBS (Gibco), 100 units/mL Antibiotic-Antimycotic (Gibco). Cells were maintained at 37°C in a 5% CO2 incubator (Binder, Tuttlingen, Germany, CB210), the media was changed twice a week. Cells were then passaged upon reaching a confluent monolayer. For this, cells were rinsed with Versene solution (Paneco, Russia) and 0.05% trypsin solution (Gibco). MSCs of the 2-3 passages were used for the experiments.

### 2.3 Adipogenic differentiation

To induce adipogenic differentiation, cells (2-3 passage) were seeded into a 24-well plate 2 days before the experiment and cultured until a confluent monolayer. For induction of adipogenic differentiation, cells were treated with DMEM Low Glucose media (containing 1.0 g/L glucose) supplemented with L-glutamine (HyClone, Cytiva, United States), 10% fetal bovine serum (FBS) (HyClone, Cytiva, United States), containing 500 units/mL Antibiotic-Antimycotic (Gibco), and adipogenic induction factors (1 µM dexamethasone (Merck, Germany), 0.5 mM 3-isobutyl-1-methylxanthine (IBMX, Millipore, United States), 10 μm/mL insulin (Paneco). For control conditions, cells were maintained in the media without differentiation inducers. Cells were cultured for 10 days. The culture media was replaced every 3 days.

To evaluate the activation of early and late adipogenic genes, cells were washed with serum-free media before initiating adipogenic differentiation. Subsequently, they were cultured in serum-free media under both standard and adipogenic conditions for 5 days before being subjected to quantitative RT-PCR analysis. Cell viability was supported by 1% BSA added to the culture media.

To test the effects of T-cadherin ligands on adipogenic differentiation, MSCs isolated from subcutaneous adipose tissue of WT and T-cadherin-deficient mice, were seeded into 24-well plates and cultured until a confluent monolayer. To exclude the effect of adiponectin and LDL present in serum, ligands were added into a serum-free media, both for control and adipogenic conditions. 1% BSA was supplemented in the culture media to maintain cell viability. Ligands were added in the following concentrations: 70 μg/mL of low-density lipoproteins (LDL) (Biorbyt, #orb1748137), or 25 μg/mL of high molecular weight adiponectin (HMW adipo) (Biovendor, #RD172023100-C), or 25 μm/mL of low molecular weight adiponectin (LMW adipo) (Abcam, #ab283926). These concentrations were previously validated in our laboratory (Balatskaya et al., 2019). Cells were cultured for 5 days. Next, live cells were stained with Nile red (Sigma Aldrich (19123-10MG) for 40 min in Hank’s buffer (Paneco) stabilized with 50 mM HEPES (Paneco) pH 7.2 at 37°C. All wells were imaged in Whole well mode using a Nikon microscope (Nikon Eclipse Ti equipped with a digital EMCCD camera Andor iXon 897 (Andor Technology, Belfast, United Kingdom), NIS-Elements 5.21.02) with red and green fluorescence registration. Cells at least in two wells per condition were analyzed in each experiment, and the experiment was repeated twice.

### 2.4 Oil red O and Nile red cell staining

The culture media was aspirated and replaced with warm Hank’s buffer (Paneco, Russia) supplemented with Hepes (Gibco) at a final concentration of 50 mM. To evaluate the accumulation of intracellular lipid droplets upon differentiation we applied Oil Red O or Nile Red staining followed by manual or automated analysis.

For Oil Red O staining, cells were fixed in 4% paraformaldehyde for 30–40 min at room temperature. Сells were rinsed 3 times (5–10 min each) with phosphate buffer saline (PBS). Oil Red O Solution (Sigma-Aldrich, United States) was added to cover the wells (500 μL–1 mL per well in a 24 well plate) for 50 min at room temperature. Oil Red O staining was visualized by Leica DMI6000B equipped with a digital camera Leica DFC7000T (Germany) and ImageJ software (NIH, Bethesda, MD, United States).

Nile Red (Sigma Aldrich, 19123-10MG) at a concentration of 2 μg/mL was added to each well for 30 min at 37°C in a 5% CO_2_ incubator. All wells of the plate were sequentially scanned using NIS-Elements (Nikon) and ImageJ software (NIH, Bethesda, MD, United States). Nile Red staining was imaged at (excitation/emission) 515 nm/590 nm for total lipids (red) and 475 nm/570 nm (emission) for non-polar lipids (green). The percentage of cells containing neutral lipids was analyzed using ImageJ software (NIH, Bethesda, MD, United States). To manually quantify the number of cells containing lipid droplets, as revealed by Oil Red O or Nile Red staining, we randomly selected 10 fields of view per well and analyzed using Nikon Eclipse Ti microscope equipped with a digital EMCCD camera Andor iXon 897 (Andor Technology, Belfast, United Kingdom. The experiment was repeated twice.

### 2.5 Rescue experiment using lentiviral constructs for T-cadherin re-expression

We utilized lentiviral constructs to determine whether re-expression of T-cadherin could restore normal adipogenic differentiation of MSCs.

For that, MSCs of the 1st passage isolated from Cdh13^ΔExon3^ and wild-type (WT) mice were seeded in 12-well plates at a density of 5 × 10^4^ cells/mL. After 12 h, cells were transduced with lentiviruses: a virus for T-cadherin re-expression (LV-LeGO-hTcad-IRES-eGFP) or control virus (LV-LeGO-eGFP). Both viruses carried a GFP tag, and after 2 days, GFP expression was detected in approximately 10% of cells. Upon reaching a confluent monolayer, the cells were induced into adipogenic differentiation. Half of the wells were supplemented with DMEM low-glucose media containing 10% FBS, 500 units/mL Antibiotic-Antimycotic (Gibco), and adipogenic induction factors, while the remaining control wells received low-glucose DMEM media, 500 units/mL Antibiotic-Antimycotic (Gibco), and 10% FBS.

After 14 days, live cells were imaged using a Leica DMI 6000B microscope (equipped with Leica DFC7000T digital camera and LAS X software) to capture GFP-expressing cells and those containing lipid droplets (phase-contrast images). Next, cells were fixed with 10% formalin, stained with DAPI (Sigma-Aldrich, D9542) for nuclei visualization, and imaged using a Nikon Eclipse Ti2 microscope equipped with a Kinetix camera (Teledyne Photometrics) in three channels: phase contrast, GFP (green fluorescence), and DAPI (blue fluorescence). Subsequently, the cells were stained with Oil Red O and re-imaged using a Nikon Eclipse Ti2 microscope equipped with a Nikon DS-Ri2 color camera to capture the phase-contrast images and red lipid droplets in transmitted light. For further image processing, automated quantification using NIS Elements AR software was applied.

To generate the LV-LeGO-hTcad-IRES-eGFP plasmid, hT-cadherin gene was amplified from pCWP1-hTcad plasmid encoding human T-cadherin cDNA (a kind gift from the Laboratory of Cellular Engineering led by T.N. Vlasik, at the Federal State Budgetary Institution National Medical Research Center of Cardiology of the Ministry of Health of the Russian Federation) using the primer pair hTcad-dir (GTC​CTC​CGA​TTG​ACT​GAG​TCG​CCC​GGA​TCC​CCC​GGA​CAA​AAT​GCA​GC) and hTcad-rev (CGG​ATC​CCA​ATT​CGA​TAT​CAA​GCT​GGT​TCA​CAG​ACA​AGC​TAA​GCT​GAA​GAG​GC); IRES-eGFP sequence was amplified from pIRES2-EGFP-p53 plasmid (Addgene plasmid #49242; http://n2t.net/addgene:49242; RRID:Addgene_49242) using the primer pair IRES2-dir (ACC​AGC​TTG​ATA​TCG​AAT​TGG​GAT​CCG) and eGFP-rev (GCT​ATA​CGA​AGT​TAT​TAG​GTC​CCT​CGA​CGT​CTA​GAT​TAC​TTG​TAC​AGC​TCG​TCC​ATG​CG). The successfully amplified fragments were isolated utilizing Cleanup Mini kit (Evrogen, Russia) and assembled using NEBuilder^®^ HiFi DNA Assembly cloning kit (NEB, United States) into BamHI-EcoRI digested LeGO-G2 vector (Addgene plasmid # 25917; https://n2t.net/addgene:25917; RRID:Addgene_25917). To generate control lentivirus (LV-LeGO-eGFP), we used LV-LeGo-G2 (https://www.addgene.org/25917/).

For lentivirus production, HEK 293 TN cells were utilized. Cells were seeded at a density of 5 × 10^4^ cells/cm^2^ in 25 cm^2^ tissue culture flasks and cultured in DMEM (Paneco, Russia) supplemented with 10% FBS (Biowest, China), and 1% penicillin/streptomycin (Paneco, Russia) at 37°C and 5% CO_2_. After 20–22 h, cells were transfected using 40 kDa linear polyethyleneimine (Polysciences, United States) at a mass ratio of 1:3 (DNA:PEI), with the following plasmids: 4 µg of vector genome; 1.3 µg of VSV-G envelope-expressing plasmid pMD2. G (Addgene plasmid # 12259; http://n2t.net/addgene:12259; RRID:Addgene_12259); and 2.6 µg of psPAX2 (Addgene plasmid # 12260; http://n2t.net/addgene:12260; RRID:Addgene_12260). The media was replaced 18–22 h post-transfection. The supernatant was collected 48 h later, clarified using a 0.45 µm filter, and stored at −80°C.

### 2.6 Automated quantification of differentiating adipocytes using neural network

For automated quantification of the ratio of differentiated adipocytes to total cells, we developed an algorithm using artificial intelligence and deep learning techniques from the NIS. ai module within the NIS Elements software package (NIS-Elements Imaging Software, 2024). In each experimental group, the cells were segmented based on the red channel of Nile Red staining using the Segment. ai neural network from the NIS. ai module of NIS Elements 5.42.02.

The Segment. ai model operates by taking an image as an input and generating a binary mask as an output, effectively segmenting the image and highlighting individual areas of interest. It allows segmentation of difficult-to-detect areas by learning on manually segmented dataset. This neural network was fine-tuned on a set of 20 images (1024 × 1024 pixels) with manually labeled masks using the Train Segment. ai function (utilizing 2,000 epochs and Dynamic Range Adaptation option).

The output of this neural network is a binary mask where each cell is segmented and isolated from its neighbors, enabling cell counting of and the identification of the cells with lipid droplets. The total number of cells was determined using the Object Count function from the General Analysis 3 module [refer to (NIS-Elements Imaging Software, 2024), GA3: an analysis pipeline with AI capabilities] NIS Elements 5.42.02 applied to the resulting mask.

To count cells with lipid droplets, droplets were segmented by thresholding the green channel of Nile Red staining, with the threshold value selected manually (consistent across all points within individual experiments). The “Having” function in the General Analysis 3 module was then employed between the masks of all cells and the mask of lipid droplets, resulting in a mask containing only cells with lipid droplets. The Object Count function from the General Analysis 3 module was again used to count these cells. Finally, the ratio was calculated by dividing the number of cells with lipid droplets by the total number of cells for each experimental group.

To further automatically quantify the results of the rescue experiment, NIS Elements AR software was used. Utilizing Oil Red O staining, DAPI staining (blue) and GFP (green) fluorescent images, the maximum intensity values of GFP and Oil Red O signals were computed for each cell. These values quantitatively reflected the proportion of virus-infected cells in the green channel (GFP fluorescence) and the accumulation of lipid droplets visualized through Oil Red O staining under transmitted light. The segmentation of each cell was carried out using the DAPI image (nuclei were detected by the Bright Spots function), followed by the cell segmentation using the Watershed function. GFP images were normalized to the highest signal intensity in the image using the Auto Contrast function, setting the most intense signal to a value of 255. To evaluate the intensity of the red signal in the Oil Red O-stained image, the formula proposed by Eggerschwiler et al. (2019) was applied: for each pixel, the value in the red channel was divided by the sum of the values in the green and blue channels; a result > 1 was considered positive. The peak intensity values of GFP and Oil Red O signals for each cell in the entire cell population were visualized using scatterplots, generated with Seaborn 0.12.2 library in Python 3.11.

### 2.7 Quantitative measurement of intracellular lipids using oil red O

Intracellular lipid quantification was conducted following the previously described methods with some modifications (Kraus et al., 2016). Cells were seeded into 24-well plates and induced for adipogenic differentiation as described above. For Oil Red staining, cells were fixed in 4% formaldehyde in PBS for 30–40 min at room temperature. Сells were carefully rinsed 3 times (5–10 min each) with phosphate buffer saline (PBS). Oil Red O Solution (Lonza, Switzerland) was added to cover the wells for 5 min at room temperature.

To quantitatively assess the Oil Red O content bound to neutral lipids within the cells, we conducted dye elution by adding 750 µL of 60% isopropanol to each well, followed by agitation for 20 min at room temperature. An additional 750 µL of 60% isopropanol was added to each well. The absorbance of the resulting eluates was measured using a Bio-Rad SmartSpec Plus spectrophotometer at a wavelength of 500 nm in Greiner Bio-One Semi-micro cuvettes (Greiner Bio-One, Germany). The obtained values were normalized to the protein content in a sample.

Protein concentration in protein extracts was determined using the Bio-Rad Protein Assay (Bio-Rad), based on the Bradford method for protein concentration evaluation. Protein content was measured on a SmartSpec Plus spectrophotometer (Bio-Rad), according to the manufacturer’s protocol, using bovine serum albumin (BSA) dilutions as standards (Bio-Rad). Protein extracts were obtained by adding 200 µL of Laemmli buffer to each well followed by a 15-min incubation.

### 2.8 Quantitative real-time polymerase chain reaction

Total RNA was extracted from murine MSCs and quantified by a NanoDrop spectrophotometer. HiPure Total RNA Plus Kit (#R411103, MAgen, China) was applied according to the manufacturer’s instruction. 500 ng of total RNA was reverse-transcribed to generate cDNA using the MMLV RT kit (Evrogen, Russia) and then subjected to real-time polymerase chain reaction (PCR) with qPCR mix-HS SYBR kit (Evrogen Russia) on a CFX96 Real-Time PCR Detection System (Bio-Rad). Primers were designed using NCBI Primer-BLAST: T-cadherin For - AAA​AAT​GCA​GCC​GAG​AAC​TCC, Rev - CCC​CCG​ACA​ATC​ACG​AGT​TC; RPLPO-13 Rev - CCC​CAG​GTA​AGC​AAA​CTT​TCT​G; For–CCCCACAAGACCAAGAGAGG. The thermal cycling program for template denaturation, primer annealing, and primer extension was 39 cycles of 95°C for 15 s, 62°C for 30 s, and 72°C for 20 s, respectively. The relative transcript level of mRNA was calculated using the 2^−ΔΔCT^ method with RPLPO-13 as a reference; normalization was done assuming as 1 the mean level of each transcript in control group (control), n = 3.

### 2.9 Western blot analysis

MSCs (WT MSCs and T−/− MSCs) of the 2st passage were seeded in 60-mm Petri dishes at a density 12-well plates at a density of 5 × 10^4^ cells/mL. Upon reaching a confluent monolayer, cells were induced into adipogenic differentiation. For that, 5 mL of DMEM media containing 1 g/L glucose, 10% FBS (Gibco), 500 units/mL Antibiotic-Antimycotic (Gibco), and adipogenic induction factors were added to the Petri dishes. Control cells were cultured in standard media. The media was replaced every 3 days. Cells were lysed on days 0, 5, 7, and 10.

Protein samples were collected by lysing the cells in a 4× Sample Buffer (Tris pH 6.8–277.8 mM, SDS - 4.4%, Glycerol - 4.3 M, β-mercaptoethanol - 10%, BromPhenol Blue - 6 mM). Samples were subjected to SDS-PAGE followed by the transfer onto Nitrocellulose (Bio Rad) or PVDF membrane (Thermo Scientific 88018) next to the protein ladder (Precision Plus Protein standard, Dual Color, BioRad #161-0374). Membranes were blocked in 5% skim milk or 5% BSA (for Leptin WB) for 12 h at + 4°C and then probed with the primary antibodies: anti-T-cadherin (rabbit polyclonal, ProSci, #3581, 1:3,000), anti-GAPDH (mouse monoclonal, sc-32233, 1:50,000), anti-Leptin (rabbit polyclonal, Abcam ab-16227, 1:3,000) and anti-Perilipin (rabbit polyclonal, Invitrogen, #PA5-55046, 1:3,000), anti-PPARγ (sc-271392 Mouse antibodies; Santa Cruz biotechnologies, United States), anti-C/EBPβ (Rabbit mAb #3082; Cell Signaling Technology Inc., United States), anti-Adiponectin (Rabbit mAb #2789; Cell Signaling Technology Inc., United States), anti-GAPDH (Rabbit antibody #5174S, Cell Signaling Technology Inc., United States). Membranes were washed to remove unbound antibodies and then incubated with horseradish antibodies peroxidase-conjugated secondary antibodies (1:8,000, Jackson ImmunoResearch, donkey anti-mouse HRP (#715–035–151), donkey anti-rabbit HRP (#111–035–003)). Protein bands were visualized using enhanced chemiluminescence with the Affinity ECL kit (Affinity, KF003) or Clarity ECL detection kit (Bio-Rad, Hercules, CA, United States) and detected with a ChemiDoc imaging system (Bio-Rad, Hercules, CA, United States) according to the manufacturer’s instructions. Image analysis was performed using Fiji software (Bethesda, NIH) or Image Lab Software (Bio-Rad, Hercules, CA, United States).

### 2.10 Enzyme-linked immunosorbent assay (ELISA)

MSCs (WT MSCs and T−/− MSCs) of the 2nd passage were seeded in 12-well plates at a density of 5 × 10^4^ cells/mL (6 wells for each cell type). Upon reaching a confluent monolayer, cells were induced into adipogenic differentiation. To this end, adipogenic media (containing 5 mL of DMEM media containing 1 g/L glucose, 10% FBS (Gibco), 500 units/mL Antibiotic-Antimycotic (Gibco), and adipogenic induction factors) were added to wells - 3 wells for each cell type. For control, cells were cultured in standard media (5 mL of DMEM media containing 1 g/L glucose, 10% FBS (Gibco), 500 units/mL Antibiotic-Antimycotic (Gibco)) - 3 wells for each cell type). Media was changed every 3 days.

To quantify the levels of secreted hormones (leptin and adiponectin), culture media from MSCs cultured in either standard or adipogenic media was collected over a 15-day period, with samples taken on days 3, 6, 9, 12, and 15. Adiponectin and leptin contents were measured using ELISA kits cat. # SEA605Mu and cat. # SEA084Mu (Cloude Clone, China), respectively. On day 15, cells were lysed in RIPA buffer (10 mM Tris-HCl, pH 7–8, 150 mM NaCl, 1% Triton X-100, 0.5% deoxycholate, 0.1% SDS) to measure intracellular perilipin-1 levels using the ELISA kit cat. # SEA745Mu (Cloude Clone, China). All assays were conducted according to the manufacturer’s instructions.

Briefly, culture media or cell lysates were added to a 96-well plate and incubated for 1 h at 37°C. Plates were incubated with primary antibodies for 1 h at 37°C. Plates were further incubated with secondary HRP antibodies for 30 min at 37°C. Bound immune complexes were visualized by adding tetramethylbenzidine (TMB) substrate and incubating in the dark for 30 min. The reaction was terminated by adding 1M H₂SO₄, and the absorbance was measured at 450 nm using a Bio-RAD ELISA plate reader. Protein concentrations were calculated based on a standard curve. Each experimental data point was analyzed in triplicate for biological replicates and in duplicate for technical replicates.

### 2.11 Statistics

Quantitative RT-PCR data were analyzed using 2-Way ANOVA with Tukey *post hoc* test. Mean values + - SD are presented.

For manual quantification of cells containing lipid droplets, the differences between the groups were assessed using the Kruskal–Wallis test followed by multiple comparisons. Statistical significance was considered at *p* < 0.05.

Quantitative evaluation of Oil Red O content was performed using two-way analysis of variance (Two-way ANOVA) followed by a *post hoc* test.

Elisa data on adiponectin and leptin secretion levels were analyzed using Repeated Measures ANOVA, while data on perilipin-1 content was analyzed with two-way ANOVA.

Unless otherwise stated, n ≥ 6.

## 3 Results

### 3.1 MSCs lacking T-cadherin are prone to spontaneous adipogenic differentiation

**FIGURE 1:**
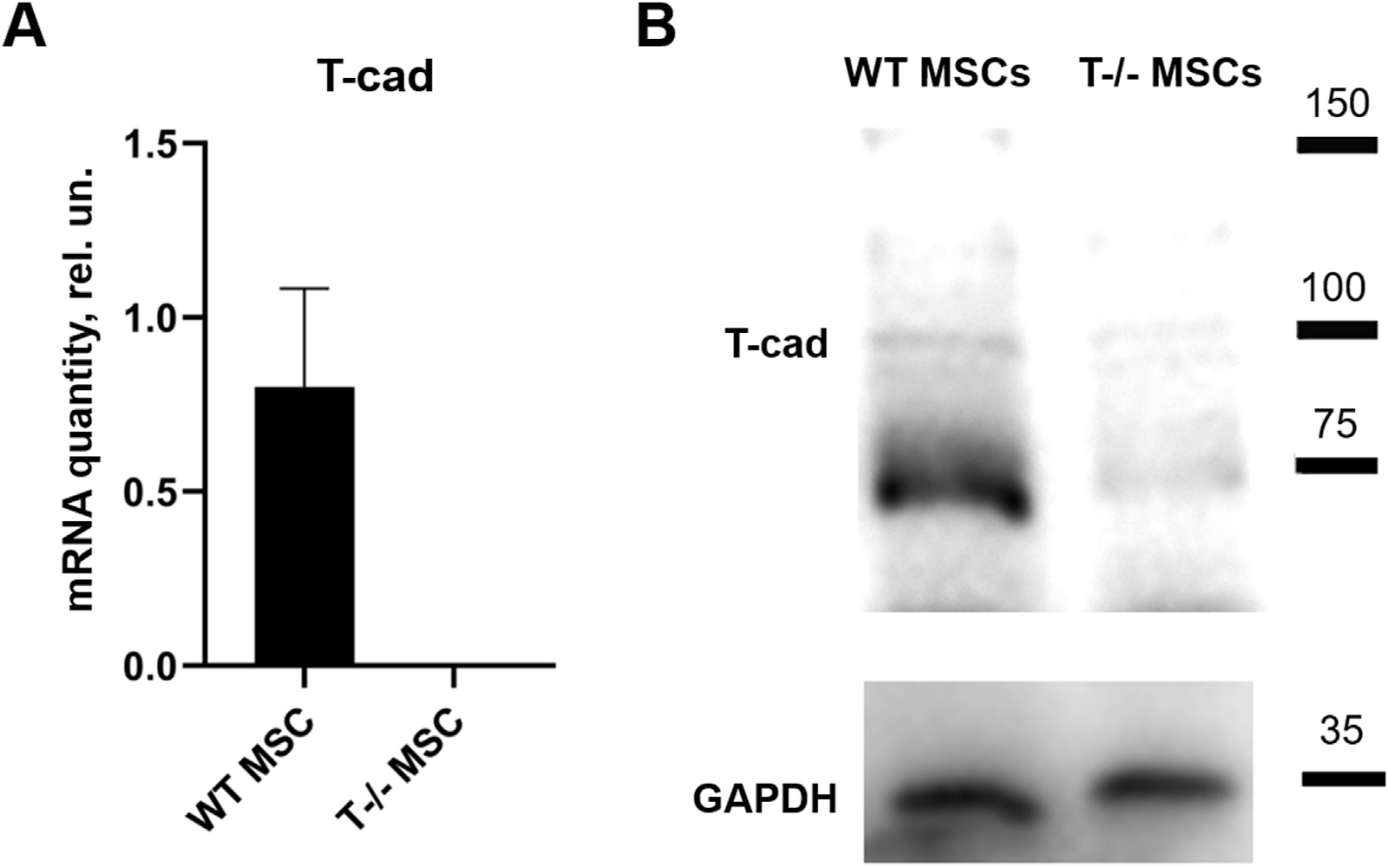
T-cadherin in content in WT MSCs and T−/− MSCs was assed using quantitative RT-PCR **(A)** and Western blot **(B)** analysis. MSCs were cultured in standard media.

Initially, RT-qPCR and Western blotting were employed to confirm the absence of full-length T-cadherin expression in T−/− MSCs (Figure 1).

**FIGURE 2:**
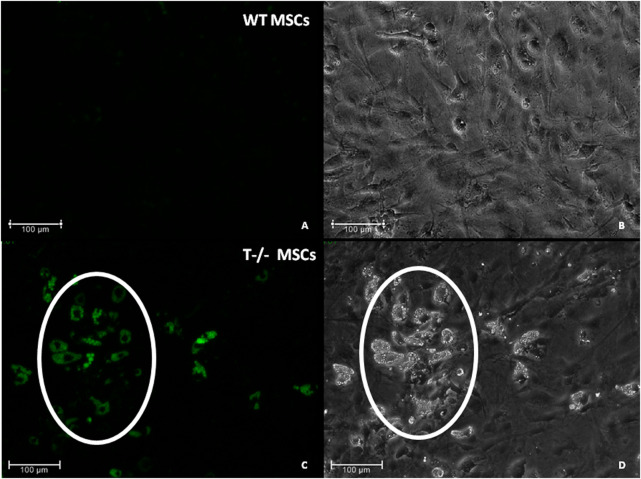
Spontaneous adipogenic differentiation in MSCs derived from murine subcutaneous adipose tissue. Upon reaching the confluent monolayer, T−/− MSCs spontaneously formed cell clusters containing lipid droplets (marked by a whit oval) [**(C)** - Nile Red staining, green fluorescence; **(D)**–phase-contrast microsopic image]. No clusters of the kind were observed in MSCs derived from control mice (WT MCSs) **(A)** (Nile Red staining) and **(B)** (phase-contrast image). Scale bar 100 µm.

To address the role of T-cadherin in adipogenic differentiation, we first isolated MSCs (WT MSCs and T−/− MSCs) from subcutaneous adipose tissue of control and T-cadherin-deficient mice and cultured the cells in standard media. We found T−/− MSCs exhibited a tendency for spontaneous adipogenic differentiation at 1–3 passages when cultured in dense monolayers under standard conditions for 4–7 days without any adipogenic inducers. To verify this phenomenon, MSCs isolated from 5 T-cadherin-deficient and 5 WT male mice (aged 8 weeks) were cultured under identical conditions. In 2 out of 5 examined samples of T−/− MSCs we detected foci containing cells with lipid droplets among the cell monolayer. Using fluorescent dye Nile Red staining we further verified the lipid accumulation in these cells. Such foci were not observed in MSCs from WT mice (Figure 2).

**FIGURE 3:**
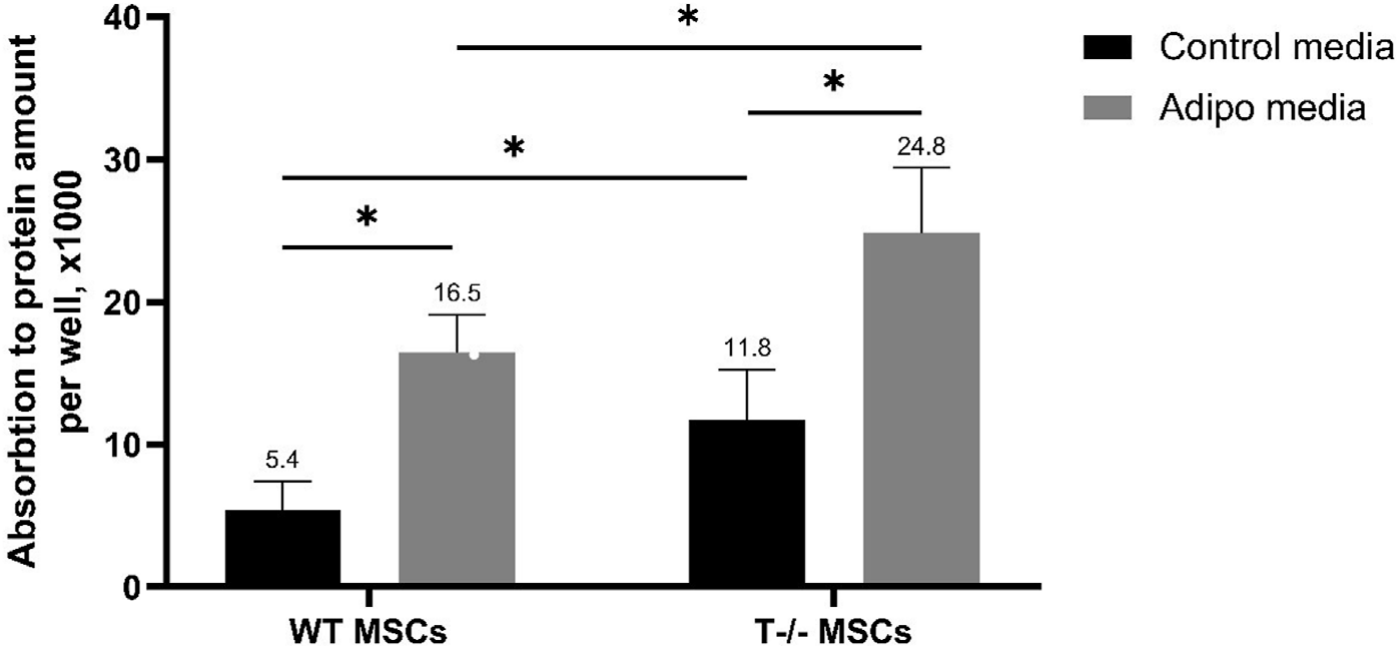
Performance of quantitative oil red O staining with arbitrary parameters. MSCs derived from WT and T−/− mice were cultured for 10 days in either control media or media for inducing adipogenic differentiation. Subsequent dye elution was performed by adding 60% isopropanol to each well. The absorbance of the eluates was measured using a Bio-Rad SmartSpec Plus spectrophotometer at a wavelength of 500 nm. The obtained values were normalized to the protein content in each sample. Quantitative evaluation was performed using two-way analysis of variance (Two-way ANOVA) followed by a *post hoc* test. Statistical significance was considered at *p* < 0.001.

Next, to explore the role of T-cadherin in adipogenic differentiation, we compared the potential of MSCs isolated from WT and T-cadherin-deficient mice to accumulate lipids in standard media or upon induction of adipogenic differentiation. For quantitative evaluation of Oil Red O content, MSCs from WT and T-cadherin-deficient mice were seeded into 12-well plates and cultured until they formed a confluent monolayer. Subsequently, cells were either induced towards the adipogenic lineage by adding the adipogenic induction factors as per the Materials and Methods section, or they were maintained in standard media for 10 days. Next, the cells were subjected to dye elution by adding 60% isopropanol to each well. The absorbance of the resulting eluates was measured and normalized to the protein content in each sample (Figure 3).

The results obtained revealed a statistically significant difference between the groups, indicating that both, the cell type (cells derived from WT or T-cadherin-deficient mice) and the media type (media for adipogenic induction or control media) independently influenced intracellular lipid accumulation. Specifically, there was a 1.5-fold increase in Oil Red O content in T−/− MSCs vs WT MSCs in adipogenic media (*P* < 0.001). Additionally, there was a 2.08-fold increase in Oil Red O content in T−/− MSCs in adipogenic media vs. T−/− MSCs in standard media (*P* < 0.001), a 2.19-fold increase in T−/− MSCs vs. WT MSCs, both in standard media (*P* < 0.05), and a 3-fold increase in Oil Red O content in WT MSCs in adipogenic media vs WT MSCs in standard media (*P* < 0.001).

Overall, MSCs derived from T-cadherin-deficient mice (T−/− MSCs) were more prone to spontaneous adipogenic differentiation and were also more susceptible to induction of adipogenic differentiation and lipid accumulation in the presence of adipogenic induction factors compared to WT MSCs.

However, the method used for quantitative evaluation of Oil Red O content does not allow for distinguishing whether there is a difference in the size of lipid droplets or in the number of cells containing them, which contribute to the overall increase in the Oil Red O staining. Specifically, individual cells may harbor large lipid droplets, or alternatively, a large number of cells may accumulate small lipid droplets.

### 3.2 T−/− MSCs accumulate large lipid droplets

**FIGURE 4:**
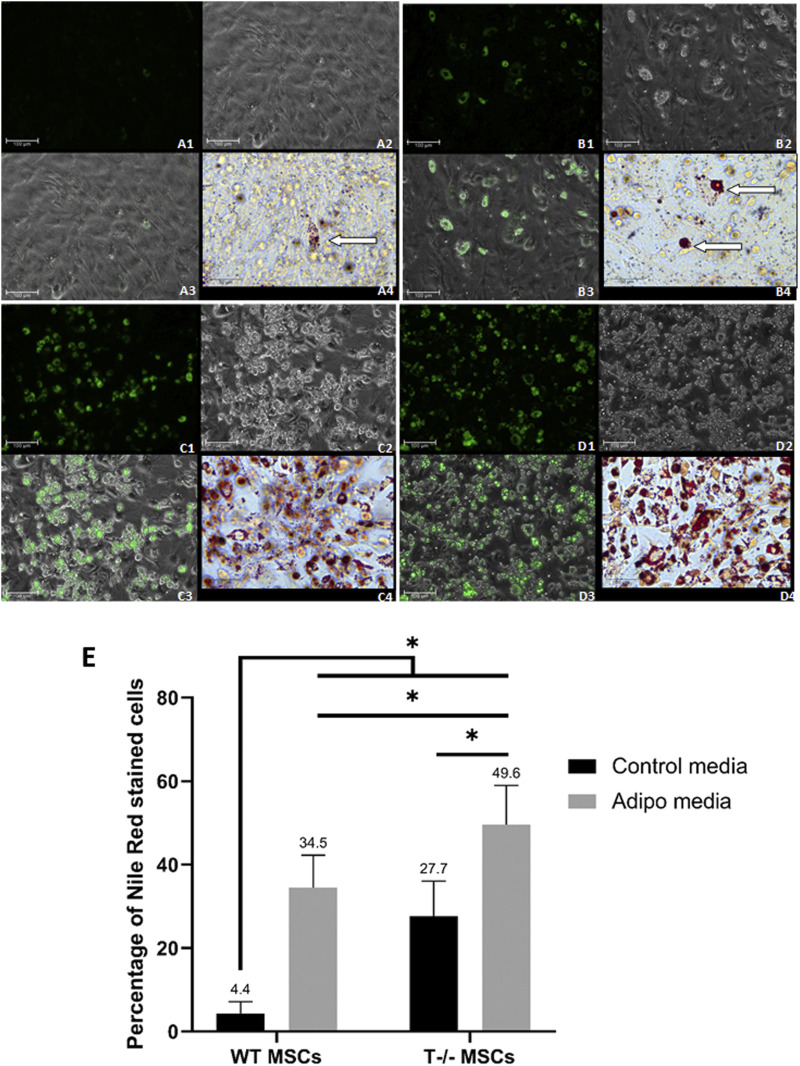
Adipogenic differentiation in MSCs derived from murine subcutaneous adipose tissue. **(A)** - WT MSCs cultured for 10 days in the standard media [**(A1)** - Nile Red, Scale bar 100 µm; **(A2)** – phase-contrast, Scale bar 100 µm; **(A3)** - phase-contrast and Nile Red merge, Scale bar 100 µm; **(A4)** - Oil Red O inset, Scale bar 50 µm] (lipid droplets marked by an arrow). **(B)** - T−/− MSCs cultured for 10 days in the standard media [**(B1)** - Nile Red, Scale bar 100 µm; **(B2)** – phase-contrast, Scale bar 100 µm; **(B3)** - phase-contrast and Nile Red merge, Scale bar 100 µm; **(B4)** - Oil Red O inset, Scale bar 50 µm] (large lipid droplets marked by arrows]. **(C)** - WT MSCs cultured for 10 days in adipogenic media [**(C1)** - Nile Red, Scale bar 100 µm; **(C2)** – phase-contrast, Scale bar 100 µm; **(C3)** - phase-contrast and Nile Red merge, Scale bar 100 µm; **(C4)** - Oil Red O inset, Scale bar 50 µm] **(D)** - T−/− MSCs cultured for 10 days in adipogenic media [**(D1)** - Nile Red, Scale bar 100 µm; **(D2)** – phase-contrast, Scale bar 100 µm; **(D3)** - phase-contrast and Nile Red merge, Scale bar 100 µm; **(D4)** - Oil Red O inset, Scale bar 50 µm]. **(E)** - Performance of quantitative Oil red O staining of T−/− MSCs and WT MSCs. The analysis was performed using 10 random fields of view. Statistical differences between the cells were assessed with Kruskal–Wallis test followed by multiple comparisons.

To further explore the role of T-cadherin in shaping lipid droplets and influencing the number of cells accumulating them, MSCs from WT and T-cadherin-deficient mice were seeded in 24-well plates and cultured until a confluent monolayer in standard media. The cells were either induced for adipogenic differentiation or maintained in standard media, both for 10 days. We performed Oil Red O and Nile Red staining combined with fluorescent, light and phase-contrast microscopy to analyze the pattern of lipid droplet accumulation in relation to T-cadherin expression (Figure 4).

Among T−/− MSCs cultured in adipogenic conditions, we observed cells with large droplets (inset in Figure 4A (4) - Oil Red O staining). WT MSCs cultured in adipogenic conditions were more homogeneous and contained small lipid droplets (inset in Figure 4B (4) - Oil Red O staining).

Single cells containing large lipid droplets were observed among T−/− MSCs cultured in standard media (inset in Figure 4C (4), Oil Red O staining). In WT MSCs cultures maintained in standard media such cells with large lipid droplets were extremely rare and mainly contained small droplets (inset in Figure 4D (4), Oil Red O staining).

To quantify the number of cells containing lipid droplets, we analyzed images obtained after Oil Red O staining of T−/− MSCs and WT MSCs after 10 days of cell culture in adipogenic conditions or standard media. We randomly selected 10 fields of view per well and calculated the percentage of cells with lipid droplets, followed by statistical analyses.

After 10 days of adipogenic differentiation, the percentage of cells with lipid droplets in both T−/− MSCs and WT MSCs significantly increased compared to standard media (a 7.8-fold increase for WT MSCs and a 1.8-fold increase T−/− MSCs). Remarkably, the percentage of these cells in T−/− MSCs was 1.4-fold higher than in WT MSCs in adipogenic conditions (Figure 4E). Statistical significance between the groups was as follows: WT MSCs in standard media vs. all other groups (*P* < 0.0001); T−/− MSCs in adipogenic condition vs. WT MSCs in adipogenic condition (a 1.4-fold increase) (*P* < 0.01); T−/− MSCs in standard media vs. T−/− MSCs in adipogenic condition (a 1.8-fold increase) (*P* < 0.001). The calculations revealed a significantly higher percentage of the cells containing lipid droplets in T−/− MSCs compared to WT MSCs, even under standard media conditions (a 6.3-fold increase) (Figure 4E).

These experiments illustrate that MSCs isolated from the subcutaneous adipose tissue of T-cadherin knockout mice exhibit an increased inclination towards adipogenic differentiation compared to MSCs from control mice. Interestingly, even in the absence of adipogenic induction factors, T−/− MSCs demonstrated a propensity to differentiate into adipocytes characterized by large lipid droplets, and this trend persisted upon the addition of adipogenic inducers.

### 3.3 Western blot analysis of early and late adipocyte differentiation markers in MSCs

To further confirm the impact of T-cadherin on adipogenic differentiation and the expression of early and late markers of adipogenesis, we performed Western blot analysis. Lysates of WT MSCs and T−/− MSCs were obtained from cells cultured in adipogenic or standard media for 10 days and samples were collected on days 0, 5, 7, and 10.

**FIGURE 5:**
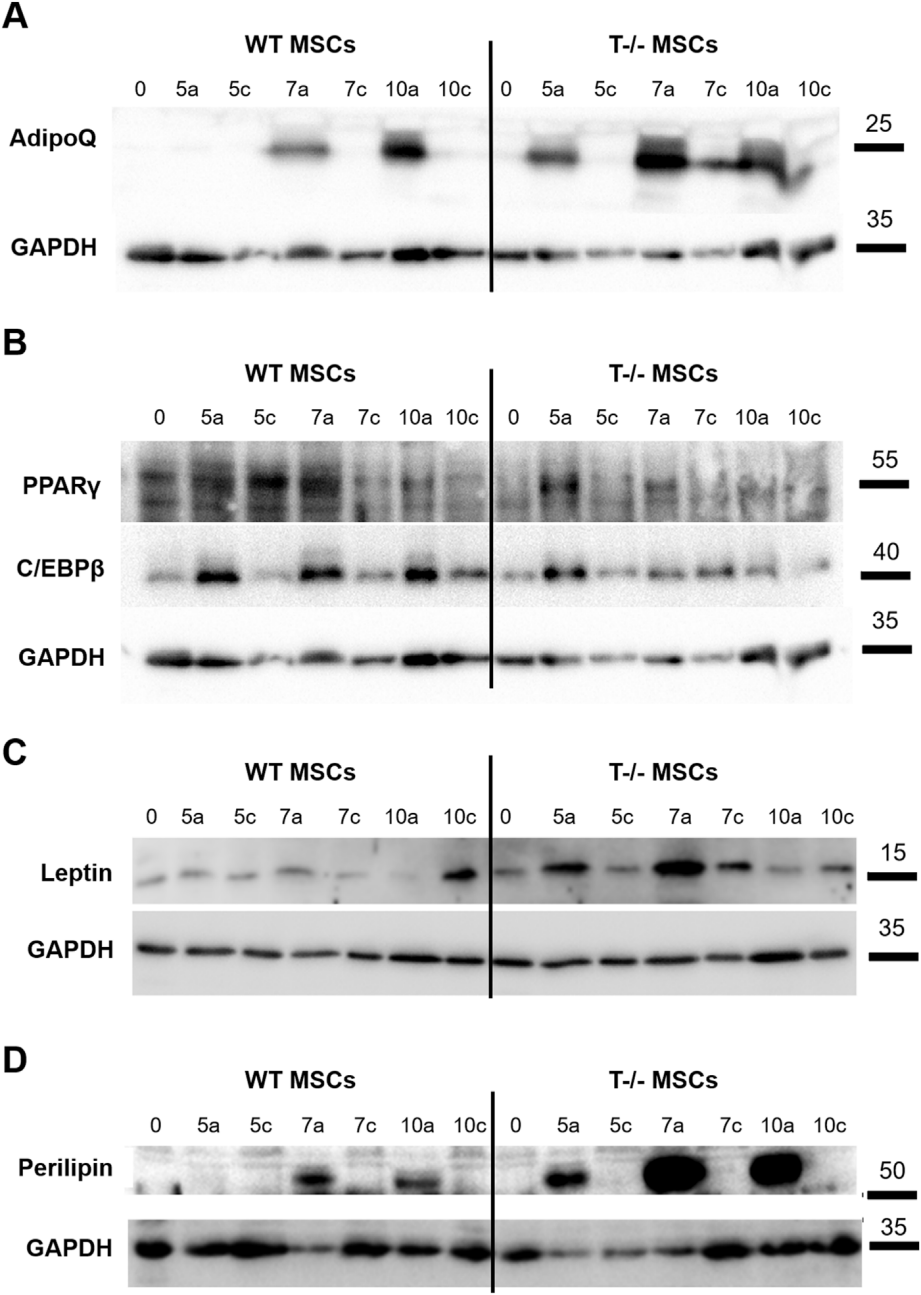
Western blot assessment of early and late adipocyte differentiation markers in lysates of WT MSCs and T−/− MSCs cultured in adipogenic (a) or standard (c) media for 10 days Commercially available antibodies were used to evaluate the protein content of PPARγ and C/EBPβ **(A)**, adiponectin **(B)**, leptin **(C)**, and perilipin **(D)**. For loading control, anti-GAPDH antibody was used. (0 – start of the experiment; 5a, 7a, 10a–days following adipogenic differentiation induction; 5c, 7c, 10c–days in the experiment where cells were maintained in media without adipogenic inducers (control).

We observed a steady rise in cellular PPARγ content in WT MSCs under adipogenic differentiation conditions up to day 7, compared to WT MSCs maintained in standard media. A similar trend was noted in T−/− MSCs, although the initial PPARγ levels in these cells were slightly lower (Figure 5A). Meanwhile, we detected a notable difference in C/EBPβ protein levels between WT MSCs and T−/− MSCs: in WT MSCs C/EBPβ protein content was elevated in cells cultured in adipogenic conditions compared to WT MSCs on days 5, 7, and 10. In contrast, the increase in C/EBPβ protein in T−/− MSCs was detected only on day 5 (Figure 5A). Given that C/EBPβ is typically upregulated during the early stages of adipogenesis, we hypothesized that T−/− MSCs progress through adipogenic differentiation at a faster rate than WT MSCs. This hypothesis was further supported by the earlier emergence of adiponectin in T−/− MSCs during adipogenic differentiation. Cellular adiponectin was detected only on the 7th day of adipogenic induction in WT MSCs, while in T−/− MSCs adiponectin was detected starting from the 5th day and was growing steadily from that moment on (Figure 5B). The Western blot analysis of leptin clearly demonstrated a consistent increase in its cellular accumulation T−/− MSCs through day 7, while in WT MSCs the results were less convincing (Figure 5C). The decrease in leptin content on the 10th day may reflect the process of its intensive secretion. Western blot analysis of leptin revealed a consistent increase in its cellular accumulation in T−/− MSCs up to day 7, whereas in WT MSCs, the results were less pronounced (Figure 5C). The observed reduction in leptin levels on day 10 in both cell types may reflect its intensified secretion processes during adipogenic differentiation. Similar dynamics were observed in perilipin protein expression: in WT MSCs, perilipin was detected on day 7 and increased further on day 10. In contrast, T−/− MSCs showed perilipin expression as early as day 5, with a dramatic rise on days 7 and 10, ultimately reaching levels that exceeded the peak expression in WT MSCs (Figure 5D).

### 3.4 ELISA analysis of adiponectin, leptin and perilipin content in MSC culture media and cell lysates

**FIGURE 6:**
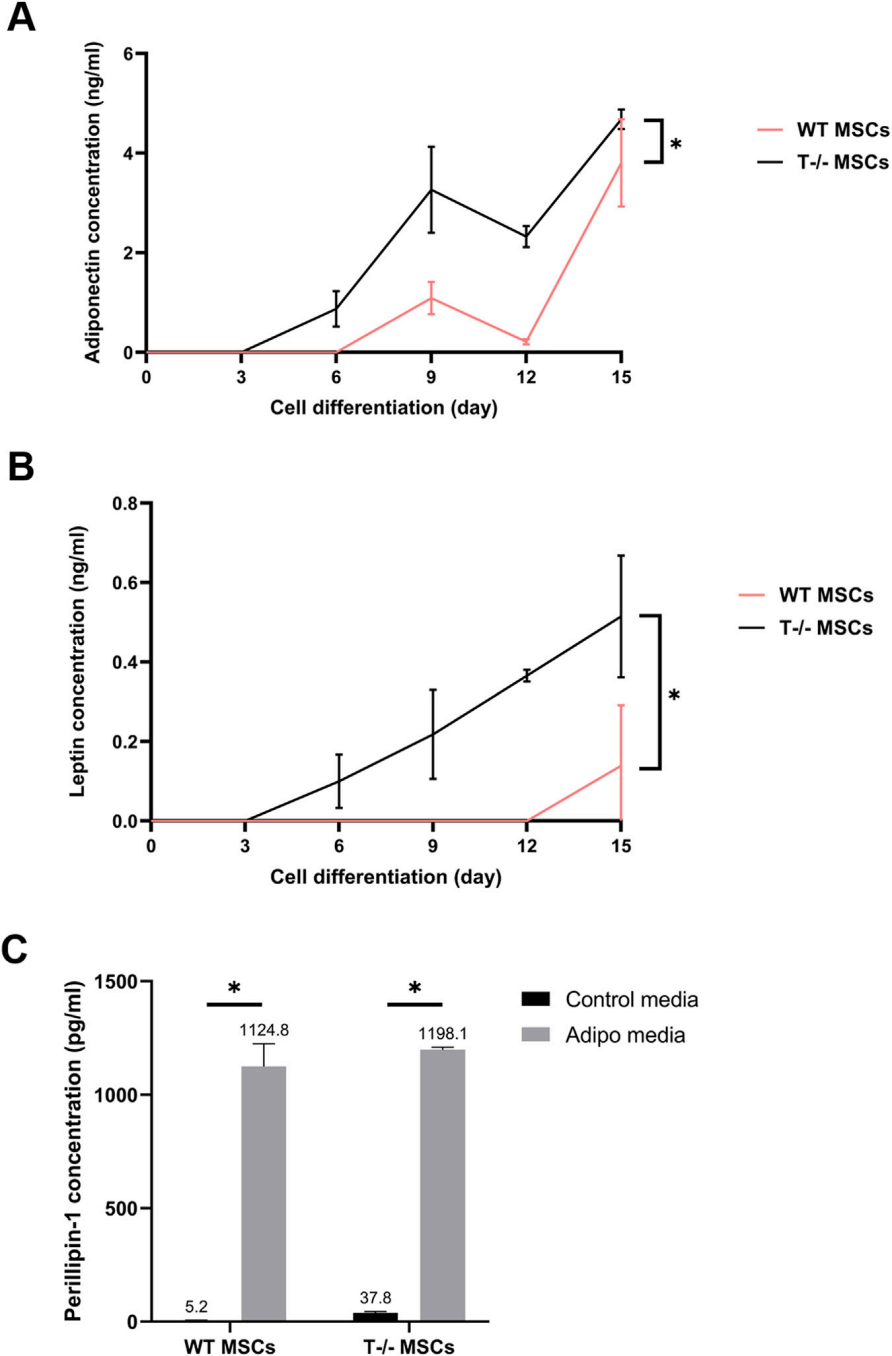
Elisa evaluation of adiponectin **(A)**, leptin **(B)**, and perilipin **(C)** in WT MSCs and T−/− MSCs cultured in adipogenic or standard media for 15 days. For adiponectin and leptin evaluation, culture media from WT MSCs and T−/− MSCs maintained in either standard or adipogenic media was collected over a 15-day period, with samples taken on days 3, 6, 9, 12, and 15. Perilipin 1 content was assessed in cell lysates obtained on the 15th day.

Given that the functional maturity of adipocytes depends on their capacity to produce and release specific adipose tissue hormones, we further analyzed the secretion of adiponectin and leptin into the culture media by MSCs. Culture media from WT MSCs and T−/− MSCs maintained in either standard or adipogenic media was collected over a 15-day period, with samples taken on days 3, 6, 9, 12, and 15. Comparative analysis of adiponectin concentration into culture media from the cells maintained in adipogenic conditions revealed that adiponectin secretion from T−/− MSCs started as early as day 3, whereas adiponectin secretion from WT MSCs could be detected only from day 6 (Figure 6A). A statistically significant difference in adiponectin secretion was observed throughout the whole experiment until day15 being much lower in WT MSCs compared to T−/− MSCs. A similar pattern was noted for leptin secretion: leptin was detected in T−/− MSCs culture media starting from day 3, whereas in WT MSCs it became detectable only from day 12. Overall, there was a statistically significant difference in leptin secretion throughout the whole experiment. Neither adiponectin nor leptin were detected in the control media (Figure 6B).

Perilipin is a lipid droplet surface protein required for lipid storage and fatty acid release (Tansey et al., 2004). Therefore, we assessed perilipin intracellular content in lysates of WT MSCs and T−/− MSCs cultured in standard and adipogenic media at the end of the experiment (day 15). Perilipin was detected in lysates from both T−/− MSCs and WT MSCs cultured in control and adipogenic media. While perilipin levels were significantly elevated in adipogenic media compared to standard media in both cell types, no difference was found between T−/− MSCs and WT MSCs (Figure 6C). These results indicate a notably elevated expression of late adipogenic markers at the protein level in T−/− MSCs compared to WT MSCs.

Overall, these findings suggest that T−/− MSCs may be predisposed to adipogenic lineage commitment, as indicated by the elevated levels of adiponectin, leptin, and perilipin in both types of cell lysates and secreted fractions.

### 3.5 Rescue experiment using lentiviral constructs for T-cadherin re-expression in MSCs

To determine whether re-expression of T-cadherin could restore typical adipogenic differentiation in MSCs, we transduced T−/− MSCs with the lentiviral construct LV-LeGO-hTcad-IRES-eGFP. For control, T−/− MSCs were transduced with LV-LeGO-eGFP lentivirus. Additionally, WT MSCs were transduced with LV-LeGO-eGFP lentivirus for internal experimental control (data not shown). To this end, MSCs of the 1st passage were seeded in 12-well plates at a density of 5 × 10^4^ cells/mL.

After 12 h, cells were transduced with lentiviruses. GFP expression was verified 2 days after transduction using fluorescent microscopy, revealing approximately 10% of cells with green fluorescence. To verify GFP expression and lipid droplet accumulation, we induced cells into adipogenic differentiation or maintained them in the same media without adipogenic inducers once they reached a confluent monolayer. After 14 days, live cells were imaged with fluorescent and phase-contrast microscopy to assess GFP expression and lipid droplet accumulation.

**FIGURE 7:**
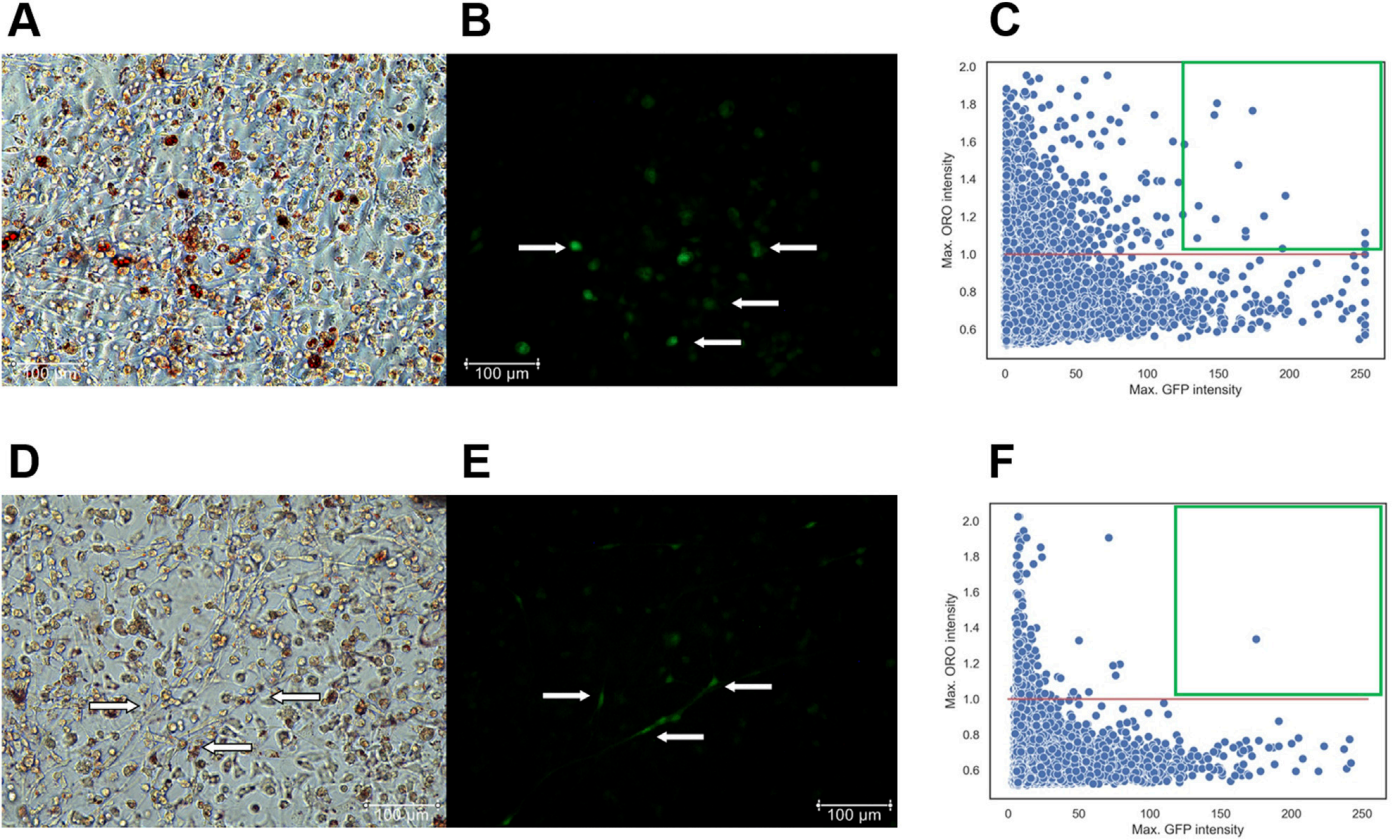
**(A–C)** - T−/− MSCs transduced with a control lentivirus construct (LV-LeGO-eGFP) and cultured for 14 days in adipogenic media. **(A)** - Oil Red O with phase-contrast; **(B)** – GFP fluorescence, Scale bar 100 µm. Cells expressing GFP are marked by arrows. **(C)** – Scatterplot illustrating the dependency of MSC differentiation efficiency on GFP fluorescence intensity. - **(D–F)** - T−/− MSCs transduced with a lentivirus construct for T-cadherin re-expression (LV-LeGO-hTcad-IRES-eGFP) and cultured for 14 days in adipogenic media. **(D)** - Oil Red O with phase-contrast; **(E)** – GFP fluorescence, Scale bar 100 µm. Cells expressing GFP are marked by arrows. **(F)** – Scatterplot illustrating the dependency of MSC differentiation efficiency on GFP fluorescence intensity. Each dot on the plot represents a single cell. The *X*-axis represents GFP fluorescence intensity. The *Y*-axis represents the maximum Oil Red O (ORO) signal, calculated using the formula (red channel value)/(blue channel value + green channel value). The red line on the *Y*-axis indicates the threshold for positive values; points above this line represents cells with lipid droplets. Green square comprises cells with a GFP signal intensity above 127 expressing the transgenes and stained for Oil Red O.

At the first stage, fixed with 10% formalin and stained with DAPI, cells were imaged to capture phase-contrast, green, and DAPI fluorescent images. At the second stage, the cells were stained with Oil Red O and re-imaged to visualize red lipid droplets under transmitted light. The NIS. ai module in NIS Elements 5.42.02 facilitated identification of the same cells imaged during the first and the second stages. Therefore, using DAPI and phase-contrast images, the neural network first recognized previously imaged cells from the first stage and identified those expressing GFP, marking viral infection. The network then overlaid these images with those obtained at the second stage, obtained after Oil Red O staining, to visualize the distribution of cells containing both lipid droplets and GFP expression (Figure 7).

For the scatterplots, we set a threshold for Oil Red O signal intensity (red line), since the cells were stained with the same reagents, allowing for a direct comparison of Oil Red O intensity. However, GFP signal intensity differed significantly between the cells transduced with LV-LeGO-hTcad-IRES-eGFP and LV-LeGO-eGFP lentiviruses, primarily due to the larger size of LV-LeGO-hTcad-IRES-eGFP construct compared to LV-LeGO-eGFP. Although this precluded the direct comparison based on GFP intensity, it still allowed for identification of transduced cells. GFP signal intensity for each cell type, transduced with either LV-LeGO-hTcad-IRES-eGFP or LV-LeGO-eGFP lentivirus, was represented within a range of 0–255. Cells on each scatterplot with a GFP signal intensity above 127 were deemed to express the transgenes, and double-positive cells (GFP and Oil Red O) were localized within the green square. In T−/− MSCs transduced with control LV-LeGO-eGFP (Figures 7A–C), we detected a significant number of double-positive cells (16 cells) in the green square (Figure 7C), indicating their adipogenic differentiation. In contrast, for T−/− MSCs transduced with LV-LeGO-hTcad-IRES-eGFP (Figures 7D–F), only one cell was detected within the green square (Figure 7F), indicating that re-expression of T-cadherin drastically reduced the cells’ ability to accumulate lipid droplets. This rescue experiment thus confirmed T-cadherin role in lipid droplet accumulation, indicating that T-cadherin re-expression suppressed lipid droplet formation.

### 3.6 Differential effects of T-cadherin ligands on adipogenic differentiation of MSCs

To investigate the potential involvement of T-cadherin in adipogenesis and explore any feedback loop from its ligands, we examined adipogenic differentiation of T−/− MSCs vs WT MSCs at the background of LDL, LMW or HMW. WT MSCs and T−/− MSCs were seeded in 24-well plates and cultured until a confluent monolayer in standard media. LMW adiponectin (25 μg/mL), HMW adiponectin (25 μg/mL) or LDL (70 μg/mL) were concurrently introduced with the cocktail of adipogenic factors into the culture media to examine their impact on the initial phase of adipogenic differentiation. Ligands were also added to standard media. To exclude the effects of adiponectin and LDL in the serum within standard culture media, cells were washed with HBSS (Paneco) prior to differentiation induction. Serum was omitted from both the adipogenic and standard media in this experiment. To maintain cell viability, 1% BSA was added. The experiment lasted for 5 days, the maximum time MSCs could endure without a loss of viability without serum.

We used 2 wells per condition: LDL, HMW adiponectin, LMW adiponectin, and without ligands in combination with either adipogenic or standard media, making a total of 8 variants for each cell type (T−/− MSCs and WT MSCs). The experiment was repeated 2 times. Live cells were stained with the fluorescent dye Nile Red in warm Hank’s solution with Hepes for 40 min at 37°C in a 5% CO_2_ incubator. Whole-field view images were captured using a Nikon microscope in both fluorescence and phase-contrast modes for subsequent analysis using a neural network for automated quantification of differentiating adipocytes.

**FIGURE 8:**
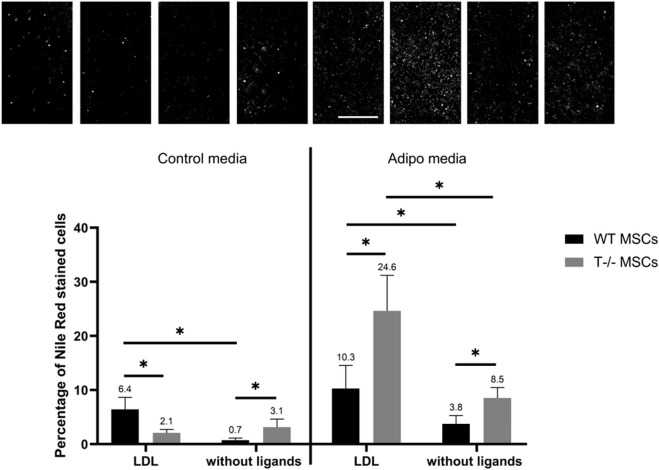
Performance of quantitative Nile Red staining after a 5-day induction in in standard media (left) or adipogenic media (right) in the presence of LDL or without ligands. Statistical differences between the cells were assessed using neural network for automated quantification with Kruskal–Wallis test followed by multiple comparisons. The top panel represents the microscopic images corresponding to each column on the graph. Scale bar 500 µm.

Adding LDL during adipogenic differentiation resulted in an almost 3-fold increase in the number of cells with lipid droplets in both WT MSCs and T−/− MSCs (Figure 8). The number of cells with lipid droplets in WT MSCs culture in adipogenic media in the presence of LDL (10.3%) was significantly higher than in adipogenic media without ligands (3.8%). (*P* < 0.0001). Similarly, the number of cells with lipid droplets in T−/− MSCs in adipogenic media in the presence of LDL (24.6%) was significantly higher than in adipogenic media without ligands (8.5%) (*P* < 0.0001). The difference between T−/− MSCs in standard media in the presence of LDL (2.1%) and standard media without ligands (3.1%) was not statistically significant (*P* = 0.1655). This notable increase in the percentage of cells with lipid droplets during adipogenic differentiation in both WT MSCs and T−/− MSCs suggests the potentiating effect of LDL on accumulation of lipid droplets. We also detected significant difference between WT MSCs (10.3%) and T−/− MSCs (24.6%) in adipogenic media in the presence of LDL (*P* < 0.05) pointing to a specific role of T-cadherin in mitigating the effects of LDL on adipogenic differentiation.

**FIGURE 9:**
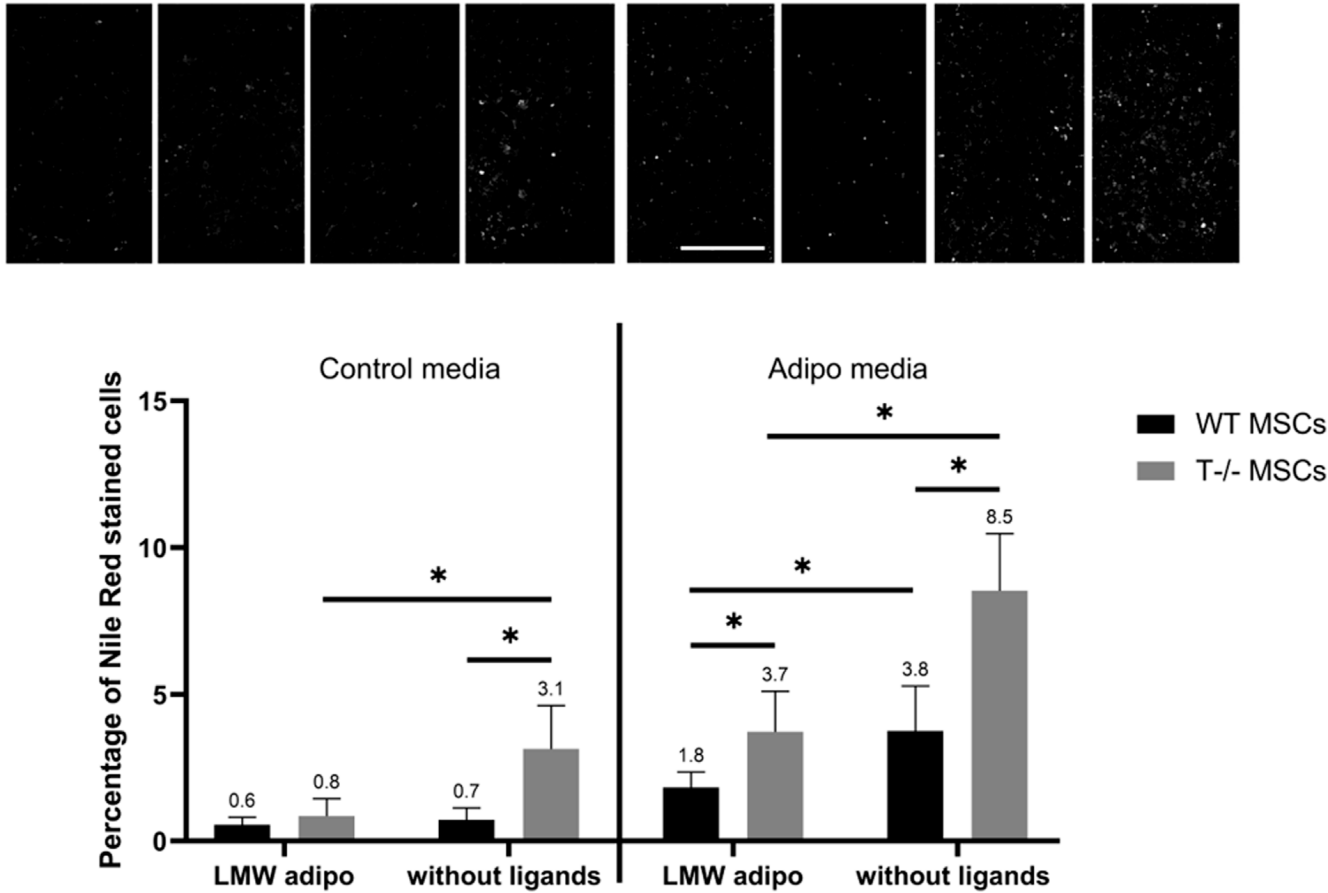
Performance of quantitative Nile Red staining after a 5-day induction in standard media (left) or in adipogenic media (right) in the presence of LMW adiponectin or without ligands. Statistical differences between the cells were assessed using neural network for automated quantification with Kruskal–Wallis test followed by multiple comparisons. The top panel represents the microscopic images corresponding to each column on the graph. Scale bar 500 µm.

The effects of LMW adiponectin on WT MSCs differentiation were revealed only in adipogenic media (Figure 9). Adding LMW adiponectin into adipogenic media resulted in a 1.2-fold decrease in the number of cells with lipid droplets (3.8% without ligands vs 1.8% in the presence of LMW adiponectin) (*P*< 00,001). However, LMW adiponectin exerted no statistically significant effect on WT MSCs in standard media (0.7% without ligands vs. 0.6% in the presence of LMW adiponectin) (*P* = 04,359). These findings imply that LMW adiponectin may have the capacity to attenuate adipogenic differentiation in MSCs under adipogenic induction conditions. In T−/− MSCs, the addition of LMW adiponectin led to a decrease in the percentage of cells containing lipid droplets in both adipogenic (8.5% without ligands vs. 3.7% in the presence of LMW adiponectin) (*P* < 0.0001) and standard media (3.1% without ligands vs. 0.8% in the presence of LMW adiponectin) (*P* < 0.0001). Specifically, there was a 2.4-fold decrease of cells with droplets in adipogenic media and a 3.8-fold decrease in standard media. These data suggest the presence of a feedback loop mediated by LMW adiponectin most likely independent of T-cadherin.

**FIGURE 10:**
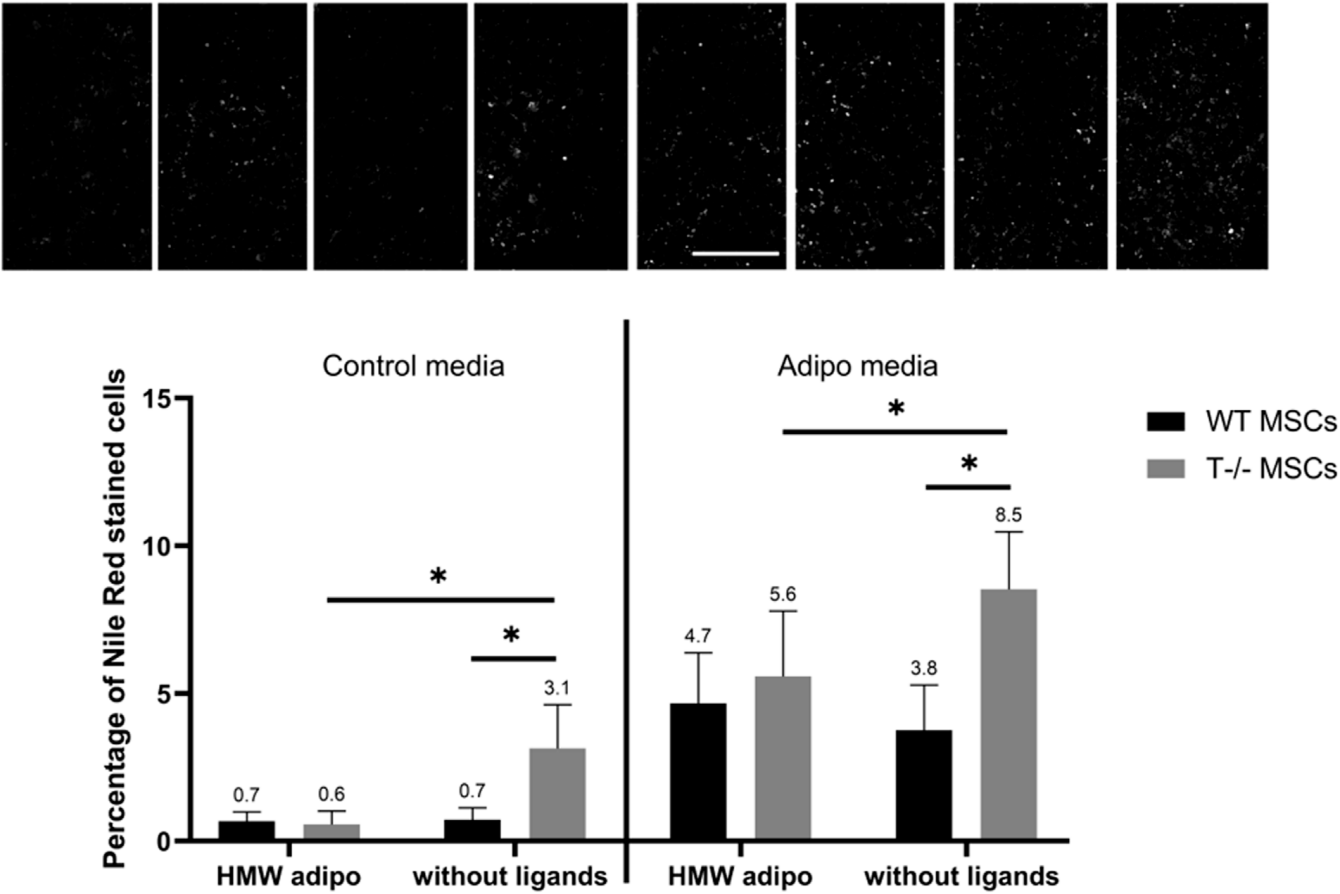
Performance of quantitative Nile Red staining after a 5-day induction in standard media (left) or in adipogenic media (right) in the presence of HMW adiponectin or without ligands. Statistical differences between the cells were assessed using neural network for automated quantification with Kruskal–Wallis test followed by multiple comparisons. The top panel represents the microscopic images corresponding to each column on the graph. Scale bar 500 µm.

Finally, adding HMW adiponectin resulted in a reduction in the percentage of cells with lipid droplets in T−/− MSCs, observed in both adipogenic and standard media (Figure 10). Specifically, there was a 1.5-fold decrease in the percentage of cells with lipid droplets (8.5% without ligands vs 5.6% in the presence of HMW adiponectin) in adipogenic media (*P* = 00,052) and a 5.2-fold decrease in standard media (3.1% without ligands vs. 0.6% in the presence of HMW adiponectin) (*P* < 00,001). However, adding HMW adiponectin did not alter the percentage of cells with lipid droplets in WT MSCs in both, standard media (0.7% without ligands vs. 0.7% in the presence of HMW adiponectin) and in adipogenic media (3.8% without ligands vs 4.7% in the presence of HMW adiponectin) (*P* = 03,527). These results indicate that T-cadherin-deficient cells are responsive to HMW adiponectin, resulting in a reduced lipid accumulation in both standard media (spontaneous differentiation) and adipogenic media. Conversely, cells expressing T-cadherin did not exhibit sensitivity to HMW adiponectin regulation.

The results of statistical analysis of the experiments presented in Figures 8–10 are shown in (Supplementary Table S1).

## 4 Discussion

Previous studies have reported the expression of T-cadherin mRNA in tissue mesenchymal progenitors from human umbilical cord perivascular cells, subcutaneous adipose tissue, and bone marrow mesenchymal stromal cells (Holley et al., 2015; Nakamura et al., 2020; Ramirez et al., 2020; Rubina et al., 2021). However, the present study marks the first investigation into the specific role of T-cadherin in adipogenic differentiation of MSCs from subcutaneous adipose tissue. Here, for the first time we present compelling evidence that T-cadherin-deficient MSCs exhibit a propensity for spontaneous adipogenic differentiation, characterized by an increased accumulation of lipid droplets (Figures 2, 3).

The sole study addressing the role of T-cadherin in adipogenesis was conducted by Göddeke et al. (2018). The authors utilized the 3T3-L1, a pre-adipocyte cell line, as an *in vitro* model for adipogenic differentiation. The highest level of T-cadherin expression was detected in undifferentiated cells, which gradually decreased during differentiation until reaching a barely detectable level in mature adipocytes. Inhibition of T-cadherin via siRNA prior to differentiation induction led to the reduced expression of key adipogenic genes (*PPARγ* and *CEBPα*) and hindered mature adipocyte formation. The authors also noted that T-cadherin played a role in lipid metabolism in progenitor cells and differentiating adipocytes, but not in mature adipocytes. Suppression of T-cadherin during adipocyte differentiation resulted in a reduced fatty acid uptake and lipid content, indicating a significant role for T-cadherin in lipid metabolism. Overall, T-cadherin was suggested to indirectly influence adipocyte differentiation, reflecting the overall health status and plasticity of adipose tissue rather than being directly involved in adipogenesis *per se*.

Our findings, as illustrated in Figures 3–6, only partially align with this research by Göddeke et al., (2018). The discrepancies may arise because 3T3-L1 cells represent committed pre-adipocyte cell line (Cave and Crowther, 2019), while MSCs constitute a heterogeneous primary culture with diverse differentiation potentials (Horwitz et al., 2005; Bäckdahl et al., 2021). Our findings, while confirming the specific role of T-cadherin played in adipogenic differentiation, also reveal substantial differences, suggesting that T-cadherin may serve as an inhibitory factor in this process. T-cadherin-deficient MSCs not only exhibited spontaneous differentiation but also manifested an increased accumulation of lipid droplets upon adipogenic induction compared to WT MSCs, as evidenced by Oil Red O and Nile Red staining, along with quantitative lipid content analysis (Figures 3, 4).

It is well acknowledged that PPARγ and CEBPα are key regulators and master genes essential for driving adipogenesis and facilitating the differentiation of preadipocytes into adipocytes (Cao et al., 1991; Moseti et al., 2016). Our Western blot analysis demonstrated a predictable and gradual increase in the expression of early adipogenic genes (PPARγ and CEBPα) in WT MSCs upon adipogenic induction starting from day 5. However, the expression patterns of PPARγ and CEBPα in T−/− MSCs were less straightforward, with only a modest elevation on day 5, followed by a subsequent decrease (Figure 5). Combined with our findings on spontaneous adipogenic differentiation (Figure 2), these results support the idea that T−/− MSCs may already be committed to adipogenic lineage, potentially progressing beyond the initial stages of PPARγ and CEBPα activation.

In addition, Western blot (Figure 5) and ELISA (Figure 6) analyses highlighted distinct patterns in the expression of late adipogenic markers. Following adipogenic induction, a notable upregulation of adiponectin content was observed in T−/− MSCs beginning on day 5, with levels steadily increasing thereafter. In contrast, adiponectin in WT MSCs was only detectable from day 7 onward. Throughout the experiment, adiponectin levels remained consistently higher in T−/− MSCs compared to WT MSCs. Therefore, these data further support the concept of T−/− MSCs adipogenic lineage commitment manifested as an early expression of adiponectin in T−/− MSCs, since adiponectin is typically detected in mature adipocytes rather than in stem/progenitor cells (Cristancho and Lazar, 2011). Similar pattern was observed for leptin, with perilipin showing an even more pronounced increase in T−/− MSCs compared to WT MSCs (Figure 5). Overall, these results indicate that T−/− MSCs produce more functionally mature adipocytes upon adipogenic induction, evidenced by a sustained elevation in adiponectin and leptin secretion and an earlier onset of hormone production.

The rescue experiment conducted using lentiviral re-expression of T-cadherin in T−/− MSCs followed by elaborate neural network analysis further confirmed the results described above and demonstrated that the initially high differentiating capacity of T−/− MSCs was suppressed by T-cadherin re-expression (Figure 7).

Dynamics of lipid droplets during adipogenesis are intricately linked to intracellular lipid storage. The quantity and size of lipid droplets vary depending on physiological or pathological stimuli encountered by the cells (Martin et al., 2005; Martin and Parton, 2006; Rizzatti et al., 2013). Our findings indicate that T−/− MSCs cultured under adipogenic conditions frequently displayed larger lipid droplets, while WT MSCs in the same conditions accumulated smaller and more homogeneous lipid droplets (insets of Figure 4). To explore the role of T-cadherin in lipid droplet formation, we further investigated how LDL, added into the culture media, affected lipid accumulation. Recently, a new concept has emerged on the regulation of glucose and cholesterol homeostasis through the interaction and signaling of insulin and LDL receptors (IR–LDLR complex) (Chandra, 2021). Accordingly, in the absence of insulin, LDLR forms a complex with IR, rendering the former functionally inactive for clearing extracellular LDL. Insulin disrupts this interaction, allowing LDLR to clear extracellular LDL (Zhu et al., 2019). This association between IR and LDLR and their dissociation by insulin may be a fundamental principle of the regulatory mechanism in normal physiological function of these receptors (Zhu et al., 2019). Our current data raise an intriguing question regarding the contribution of T-cadherin in this signaling pathway. In standard media, administration of LDL led to a modest yet statistically significant rise in lipid droplet accumulation in WT MSCs, while exerting no effect on T−/− MSCs. Meanwhile, upon adipogenic induction with a mixture of factors including insulin, both WT MSCs and T−/− MSCs exhibited an increased lipid droplet accumulation in response to LDL, suggesting a potential dissociation of IR–LDLR complex. However, T-cadherin expression in WT MSCs notably attenuated the stimulatory effects of LDL on intracellular lipid accumulation compared to T−/− MSCs (Figure 8). These data suggest that T-cadherin may operate in a signaling complex with LDLR or IR–LDLR, thus modulating their activity or downstream signaling as has been previously proposed by our group (Rubina et al., 2021).

Another explanation for our results is that T-cadherin present on the cell surface may act as a trap for LDL attenuating their impact on lipid droplet accumulation. In line with this are our previously published data indicating a progressive elevation in T-cadherin expression on both endothelial cells and pericytes with the advancement of atherosclerosis (Ivanov et al., 2001), suggesting a protective role for T-cadherin. Of note, pericytes have much in common with cultured MSCs (da Silva Meirelles et al., 2024), thus potentially implicating the same signaling pathways in both cell types.

In contrast, other published data reported inhibitory effects of LDL on adipogenesis. Cyr et al. (2021), utilizing Simpson-Golabi Behmel-syndrome (SGBS) cells as preadipocytes derived from human white adipose tissue biopsies, demonstrated that human LDL in adipogenic media impaired differentiation and function of these cells in a dose-dependent manner. Furthermore, chronic exposure to native LDL inhibited adipocyte function *in vitro* measured as reduced lipoprotein lipase activity. Native LDL also suppressed expression of adipogenic genes, such as *CEBPa*, although not affecting *PPARγ*, as well as genes associated with lipid metabolism (*LPL*, *LDLR*, and *CD36*, *ADIPOQ,* etc.), glucose metabolism and insulin action. Conversely, the genes linked to inflammation, such as *MCP-1* and *IL-1β*, were upregulated (Cyr et al., 2021; Bissonnette et al., 2023). The authors further suggested that increased LDL uptake by preadipocytes in adipose tissue, coupled with an elevated expression of LDLR and CD36 (scavenger receptor class B member 3), could potentially impair white adipose tissue function. This scenario may subsequently trigger macrophage infiltration into adipose tissue and disrupt adipocyte renewal. The disparities observed in the effects of LDL between the studies by Cyr et al. (2021) and our current research could be attributed to various cell models used. In the future, exploring the signaling pathways triggered during adipogenic differentiation in the presence of LDL, or elucidating the variances in gene activation based on the presence or absence of T-cadherin, could offer valuable insights.

Next, we evaluated the effects of LMW and HMW adiponectin on adipogenic differentiation using T−/− MSCs and WT MSCs to unveil the role of T-cadherin in mediating a potential feedback loop from this adipokine (Figures 6, 7). Generally, adiponectin is considered to encourage a ‘‘healthy’’ expansion of adipose tissue (Stern et al., 2016). Numerous studies have shown that adiponectin improves insulin sensitivity (Berg et al., 2001) and triggers lipid accumulation within the adipose tissue, rescuing ectopic lipid accumulation in animal models (Yamauchi et al., 2002; Xu et al., 2003). In line with this, adiponectin overexpression in 3T3-L1 cells was reported to stimulate adipocyte differentiation and lipid accumulation *in vitro* (Fu et al., 2005). Adiponectin-overexpressing cells demonstrated a more sustained and elevated gene expression of adipogenic transcription factors, such as *C/EBP2*, *PPARs*, and adipocyte determination and differentiation factor 1/sterol-regulatory element binding protein 1c (*ADD1/SREBP1c*). Moreover, mature adipocytes, overexpressing adiponectin, had a heightened lipid accumulation and insulin-responsive glucose transport as compared with control cells, as well as accumulated larger and more numerous lipid droplets. The authors suggested that adiponectin operates as an autocrine factor stimulating cell proliferation and differentiation in adipose tissue (Fu et al., 2005). Yet, another study on chicken showed that adiponectin negatively regulates the expression of C/EBPα and fatty acid synthase accompanied by a decrease in adipocyte differentiation as measured by Oil Red O staining (Yan et al., 2014). Using a transgenic mouse model allowing overexpression of native full-length adiponectin in white adipose tissue, Bauche et al. (2019) reported the impairment of adipocyte differentiation *in vivo* contributing to the anti-adiposity effect of adiponectin. These authors also demonstrated that recombinant full-length adiponectin added to the culture media of differentiating 3T3-F442A adipocytes downregulated *C/EBPa* in *in vitro* experiments (Bauche et al., 2007). However, since the authors of these studies did not specify the exact form of adiponectin utilized, the disparities may stem from the preferential binding of different forms of adiponectin to specific receptors and activating distinct intracellular signaling pathways. For instance, AdipoR1 receptor has a higher affinity for LMW adiponectin, while the AdipoR2 receptor preferentially binds the HMW adiponectin. Through binding to AdipoR1 and AdipoR2, adiponectin activates AMPK and PPARα, correspondingly (Nguyen, 2020).

The novelty of the current study lies in its comparative approach, which aims to assess and contrast the effects of different forms of adiponectin, LMW adiponectin and HMW adiponectin. While in standard media LMW adiponectin decreased the number of cells in T−/− MSCs, with no effect on WT MSCs, in adipogenic conditions LMW adiponectin suppressed lipid accumulation in both cell types (Figure 9). These effects are likely independent of T-cadherin, consistent with the fact that T-cadherin serves as the exclusive receptor for HMW adiponectin (Hug et al., 2004; Fukuda et al., 2017). In contrast, HMW adiponectin suppressed lipid accumulation in T−/− MSCs in both standard and adipogenic media (Figure 10). These data suggest the involvement of T-cadherin in HMW adiponectin-mediated adipogenesis, yet the exact mechanism behind this phenomenon warrants further investigation.

Adiponectin is well-known for its role in targeting the metabolically active tissues, including liver, muscle, and adipose tissue, upon its release from adipocytes into the bloodstream. It enhances insulin signaling, facilitating glucose uptake, suppressing glucose production in the liver and muscle, and promoting fatty acid oxidation (Nguyen, 2020). Furthermore, adiponectin stimulates ceramidase activation in a AdipoRs-dependent way, leading to the reduced intracellular and circulating levels of ceramide. This process improves insulin sensitivity by disrupting ceramide accumulation and decreasing apoptosis via the formation of sphingosine-1-phosphate (S1P) (Luo and Liu, 2022). Importantly, Obata et al. (2018), demonstrated that adiponectin undergoes endocytosis in a T-cadherin-related manner, yet independent of AdipoRs, and accumulates in multivesicular bodies (MVBs) - the primary site of exosome generation. Subsequently, both adiponectin and T-cadherin are released as exosomal cargo and T-cadherin enhances exosome production from T-cadherin–expressing endothelial cells in mouse aorta *in vivo* and *in vitro.* These effects were not detected in T-cadherin-deficient mice or cells. These findings, along with our present data indicating the inhibitory effect of HMW adiponectin on lipid accumulation, emphasize the potential importance of T-cadherin as a regulator of adiponectin-dependent insulin sensitivity, suggesting that the reduction of cellular ceramides could be one of the underlying mechanisms.

To infer, here we have demonstrated for the first time the role of T-cadherin in MSCs adipogenic differentiation and accumulation of lipid droplets. Depending on the presence of T-cadherin, distinct morphological characteristics emerged. The absence of T-cadherin facilitated the spontaneous adipogenic differentiation of MSCs, leading to the formation of large lipid droplets in the cytoplasm. This trend persisted upon adipogenic induction with a differentiating cocktail. Along with these results, our rescue experiments along with Western blot and Elisa data further support the concept that MSCs lacking T-cadherin expression are predisposed towards the adipogenic lineage. Furthermore, we conducted an original comparative analysis examining the effects of two well-known T-cadherin ligands, LDL and adiponectin, on lipid droplet accumulation. Our findings revealed their differential effects: LDL stimulated adipogenic differentiation, while T-cadherin expression appeared to mitigate the impact of LDL on lipid droplet accumulation. Additionally, we investigated the effects of both LMW and HMW adiponectin on lipid droplet accumulation in relation to T-cadherin expression. Interestingly, we have found that LMW adiponectin suppressed lipid droplet accumulation, but this effect was most likely independent of T-cadherin. Conversely, the absence of T-cadherin rendered cells more susceptible to the suppressive effects of HMW adiponectin on adipogenesis.

In conclusion, the potential crosstalk between T-cadherin and other receptors remains largely unclear. This leaves room for the possibility that T-cadherin may either activate or modulate distinct signaling pathways through lateral interactions with LDLR and AdipoRs, as suggested in our recently published review (Rubina et al., 2021). Further investigation is needed to elucidate the underlying molecular mechanisms of this interplay.

## Data Availability

The original contributions presented in the study are included in the article/Supplementary Material, further inquiries can be directed to the corresponding author.
