# Supplementary material for: T-cadherin modulates adipogenic differentiation in mesenchymal stem cells: insights into ligand interactions
Source: Front Cell Dev Biol. 2024 Dec 9;12:1446363. doi: 10.3389/fcell.2024.1446363 (PMC11663858; doi:10.3389/fcell.2024.1446363)
Supplement: Supplementary file 1 [file DataSheet1.docx]

Supplementary Material

# Supplementary Data

To investigate the potential involvement of T-cadherin in adipogenesis, we examined adipogenic differentiation of T-/- MSCs vs. WT MSCs in the presence of LDL, LMW and HMW adiponectin. WT MSCs and T-/- MSCs were seeded in 24-well plates. LMW adiponectin (25 µg/ml), HMW adiponectin (25 µg/ml) or LDL (70 µg/ml) were simultaneously introduced with the cocktail of adipogenic factors into the culture media. Ligands were also added to the cell in the standard media. Serum was omitted from both the adipogenic and standard media. To sustain cell viability, 1% BSA was added instead. The experiment lasted for 5 days (Figure S1).


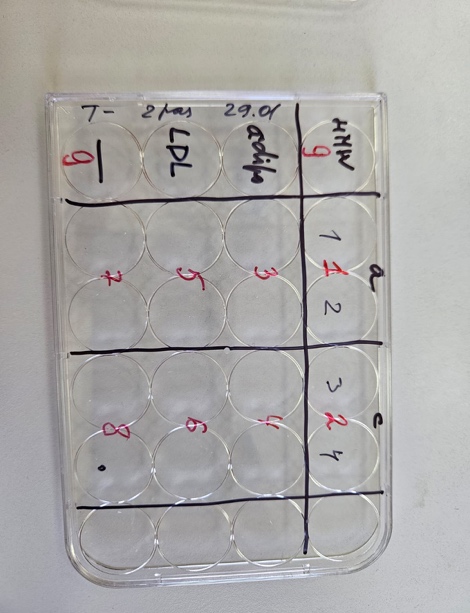

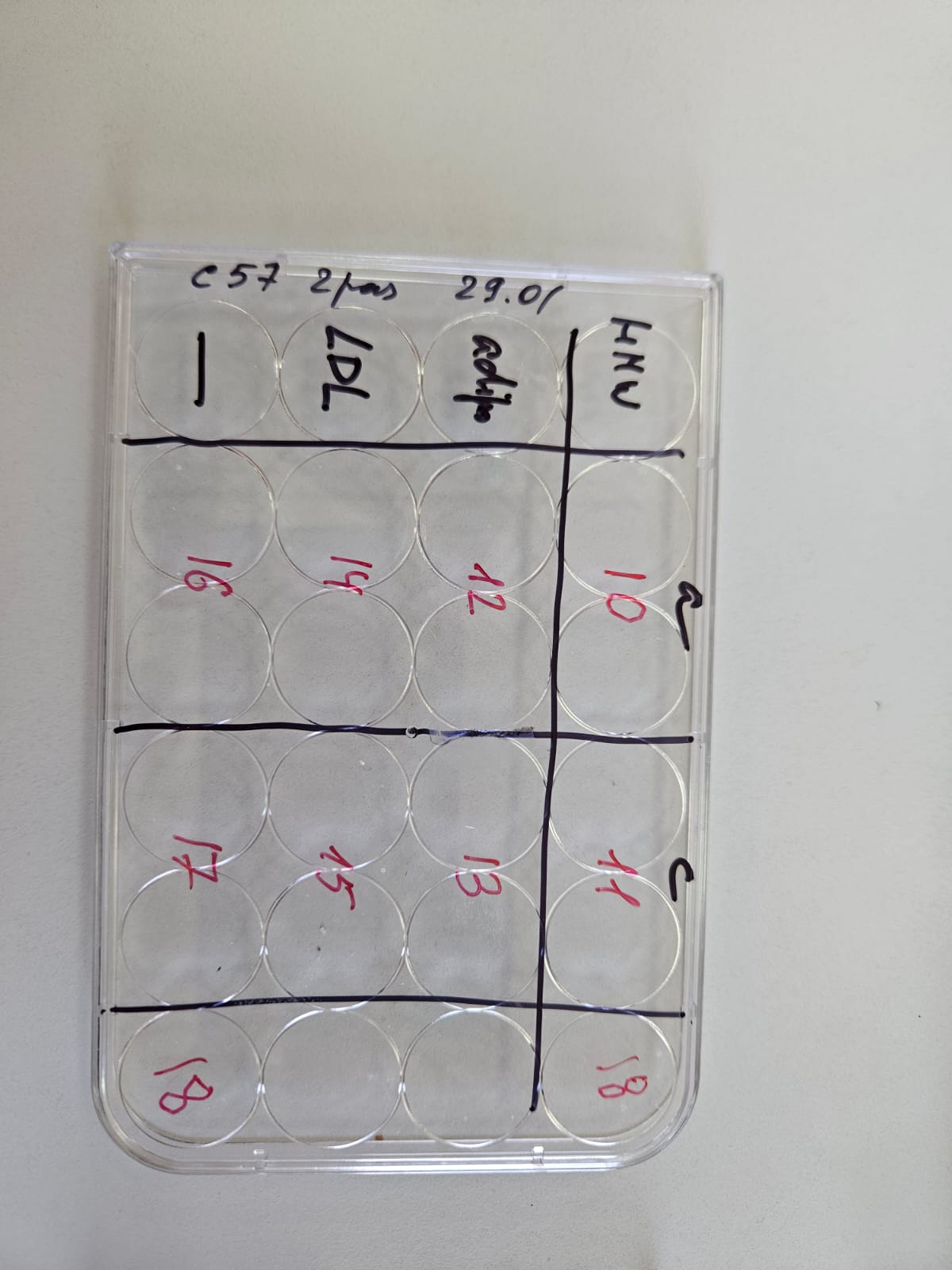


**Figure S1.** Representative photo of a 24-well plate with T-/- MSCs and WT MSCs in the standard or adipogenic media with addition of T-cadherin ligands (LDL, LMW and HMW adiponectin) or without the ligands.

**Table S1.** The differences between the groups were assessed using the Mann–Whitney U test. Statistical significance was considered at p < 0.05.

| MSCs | Media | Ligand | **VS** | MSCs | Media | Ligand | P value |
| --- | --- | --- | --- | --- | --- | --- | --- |
| WT | control | LMW adipo |  | WT | control | HMW adipo | 0,4359 |
| WT | control | LMW adipo |  | WT | control | without ligand | 0,4359 |
| WT | control | LMW adipo |  | T-/- | control | HMW adipo | 0,6842 |
| WT | control | LMW adipo |  | T-/- | control | LMW adipo | 0,1655 |
| WT | control | LMW adipo |  | WT | adipo | LMW adipo | <0,0001 |
| WT | control | LMW adipo |  | T-/- | control | without ligand | <0,0001 |
| WT | control | LMW adipo |  | T-/- | control | LDL | <0,0001 |
| WT | control | LMW adipo |  | WT | control | LDL | <0,0001 |
| WT | control | LMW adipo |  | WT | adipo | without ligand | <0,0001 |
| WT | control | LMW adipo |  | T-/- | adipo | LMW adipo | <0,0001 |
| WT | control | LMW adipo |  | WT | adipo | HMW adipo | <0,0001 |
| WT | control | LMW adipo |  | T-/- | adipo | HMW adipo | <0,0001 |
| WT | control | LMW adipo |  | T-/- | adipo | without ligand | <0,0001 |
| WT | control | LMW adipo |  | WT | adipo | LDL | <0,0001 |
| WT | control | LMW adipo |  | T-/- | adipo | LDL | <0,0001 |
| WT | control | HMW adipo |  | WT | control | without ligand | 0,8534 |
| WT | control | HMW adipo |  | T-/- | control | HMW adipo | 0,3527 |
| WT | control | HMW adipo |  | T-/- | control | LMW adipo | 0,6305 |
| WT | control | HMW adipo |  | WT | adipo | LMW adipo | <0,0001 |
| WT | control | HMW adipo |  | T-/- | control | without ligand | <0,0001 |
| WT | control | HMW adipo |  | T-/- | control | LDL | <0,0001 |
| WT | control | HMW adipo |  | WT | control | LDL | <0,0001 |
| WT | control | HMW adipo |  | WT | adipo | without ligand | <0,0001 |
| WT | control | HMW adipo |  | T-/- | adipo | LMW adipo | <0,0001 |
| WT | control | HMW adipo |  | WT | adipo | HMW adipo | <0,0001 |
| WT | control | HMW adipo |  | T-/- | adipo | HMW adipo | <0,0001 |
| WT | control | HMW adipo |  | T-/- | adipo | without ligand | <0,0001 |
| WT | control | HMW adipo |  | WT | adipo | LDL | <0,0001 |
| WT | control | HMW adipo |  | T-/- | adipo | LDL | <0,0001 |
| WT | control | without ligand |  | T-/- | control | HMW adipo | 0,3150 |
| WT | control | without ligand |  | T-/- | control | LMW adipo | 0,6305 |
| WT | control | without ligand |  | WT | adipo | LMW adipo | <0,0001 |
| WT | control | without ligand |  | T-/- | control | without ligand | <0,0001 |
| WT | control | without ligand |  | T-/- | control | LDL | <0,0001 |
| WT | control | without ligand |  | WT | control | LDL | <0,0001 |
| WT | control | without ligand |  | WT | adipo | without ligand | <0,0001 |
| WT | control | without ligand |  | T-/- | adipo | LMW adipo | <0,0001 |
| WT | control | without ligand |  | WT | adipo | HMW adipo | <0,0001 |
| WT | control | without ligand |  | T-/- | adipo | HMW adipo | <0,0001 |
| WT | control | without ligand |  | T-/- | adipo | without ligand | <0,0001 |
| WT | control | without ligand |  | WT | adipo | LDL | <0,0001 |
| WT | control | without ligand |  | T-/- | adipo | LDL | <0,0001 |
| T-/- | control | HMW adipo |  | T-/- | control | LMW adipo | 0,3150 |
| T-/- | control | HMW adipo |  | WT | adipo | LMW adipo | <0,0001 |
| T-/- | control | HMW adipo |  | T-/- | control | without ligand | <0,0001 |
| T-/- | control | HMW adipo |  | T-/- | control | LDL | <0,0001 |
| T-/- | control | HMW adipo |  | WT | control | LDL | <0,0001 |
| T-/- | control | HMW adipo |  | WT | adipo | without ligand | <0,0001 |
| T-/- | control | HMW adipo |  | T-/- | adipo | LMW adipo | <0,0001 |
| T-/- | control | HMW adipo |  | WT | adipo | HMW adipo | <0,0001 |
| T-/- | control | HMW adipo |  | T-/- | adipo | HMW adipo | <0,0001 |
| T-/- | control | HMW adipo |  | T-/- | adipo | without ligand | <0,0001 |
| T-/- | control | HMW adipo |  | WT | adipo | LDL | <0,0001 |
| T-/- | control | HMW adipo |  | T-/- | adipo | LDL | <0,0001 |
| T-/- | control | LMW adipo |  | WT | adipo | LMW adipo | <0,0001 |
| T-/- | control | LMW adipo |  | T-/- | control | without ligand | <0,0001 |
| T-/- | control | LMW adipo |  | T-/- | control | LDL | <0,0001 |
| T-/- | control | LMW adipo |  | WT | control | LDL | <0,0001 |
| T-/- | control | LMW adipo |  | WT | adipo | without ligand | <0,0001 |
| T-/- | control | LMW adipo |  | T-/- | adipo | LMW adipo | <0,0001 |
| T-/- | control | LMW adipo |  | WT | adipo | HMW adipo | <0,0001 |
| T-/- | control | LMW adipo |  | T-/- | adipo | HMW adipo | <0,0001 |
| T-/- | control | LMW adipo |  | T-/- | adipo | without ligand | <0,0001 |
| T-/- | control | LMW adipo |  | WT | adipo | LDL | <0,0001 |
| T-/- | control | LMW adipo |  | T-/- | adipo | LDL | <0,0001 |
| WT | adipo | LMW adipo |  | T-/- | control | without ligand | 0,0232 |
| WT | adipo | LMW adipo |  | T-/- | control | LDL | 0,4359 |
| WT | adipo | LMW adipo |  | WT | control | LDL | <0,0001 |
| WT | adipo | LMW adipo |  | WT | adipo | without ligand | 0,0039 |
| WT | adipo | LMW adipo |  | T-/- | adipo | LMW adipo | 0,0001 |
| WT | adipo | LMW adipo |  | WT | adipo | HMW adipo | <0,0001 |
| WT | adipo | LMW adipo |  | T-/- | adipo | HMW adipo | <0,0001 |
| WT | adipo | LMW adipo |  | T-/- | adipo | without ligand | <0,0001 |
| WT | adipo | LMW adipo |  | WT | adipo | LDL | <0,0001 |
| WT | adipo | LMW adipo |  | T-/- | adipo | LDL | <0,0001 |
| T-/- | control | without ligand |  | T-/- | control | LDL | 0,1655 |
| T-/- | control | without ligand |  | WT | control | LDL | 0,0011 |
| T-/- | control | without ligand |  | WT | adipo | without ligand | 0,4359 |
| T-/- | control | without ligand |  | T-/- | adipo | LMW adipo | 0,2799 |
| T-/- | control | without ligand |  | WT | adipo | HMW adipo | 0,0524 |
| T-/- | control | without ligand |  | T-/- | adipo | HMW adipo | 0,0185 |
| T-/- | control | without ligand |  | T-/- | adipo | without ligand | <0,0001 |
| T-/- | control | without ligand |  | WT | adipo | LDL | <0,0001 |
| T-/- | control | without ligand |  | T-/- | adipo | LDL | <0,0001 |
| T-/- | control | LDL |  | WT | control | LDL | <0,0001 |
| T-/- | control | LDL |  | WT | adipo | without ligand | <0,0001 |
| T-/- | control | LDL |  | T-/- | adipo | LMW adipo | 0,0007 |
| T-/- | control | LDL |  | WT | adipo | HMW adipo | <0,0001 |
| T-/- | control | LDL |  | T-/- | adipo | HMW adipo | <0,0001 |
| T-/- | control | LDL |  | T-/- | adipo | without ligand | <0,0001 |
| T-/- | control | LDL |  | WT | adipo | LDL | <0,0001 |
| T-/- | control | LDL |  | T-/- | adipo | LDL | <0,0001 |
| WT | control | LDL |  | WT | adipo | without ligand | 0,0029 |
| WT | control | LDL |  | T-/- | adipo | LMW adipo | 0,0029 |
| WT | control | LDL |  | WT | adipo | HMW adipo | 0,0753 |
| WT | control | LDL |  | T-/- | adipo | HMW adipo | 0,1230 |
| WT | control | LDL |  | T-/- | adipo | without ligand | 0,0185 |
| WT | control | LDL |  | WT | adipo | LDL | 0,0115 |
| WT | control | LDL |  | T-/- | adipo | LDL | <0,0001 |
| WT | adipo | without ligand |  | T-/- | adipo | LMW adipo | 0,5288 |
| WT | adipo | without ligand |  | WT | adipo | HMW adipo | 0,3527 |
| WT | adipo | without ligand |  | T-/- | adipo | HMW adipo | 0,0433 |
| WT | adipo | without ligand |  | T-/- | adipo | without ligand | <0,0001 |
| WT | adipo | without ligand |  | WT | adipo | LDL | <0,0001 |
| WT | adipo | without ligand |  | T-/- | adipo | LDL | <0,0001 |
| T-/- | adipo | LMW adipo |  | WT | adipo | HMW adipo | 0,1051 |
| T-/- | adipo | LMW adipo |  | T-/- | adipo | HMW adipo | 0,0232 |
| T-/- | adipo | LMW adipo |  | T-/- | adipo | without ligand | <0,0001 |
| T-/- | adipo | LMW adipo |  | WT | adipo | LDL | <0,0001 |
| T-/- | adipo | LMW adipo |  | T-/- | adipo | LDL | <0,0001 |
| WT | adipo | HMW adipo |  | T-/- | adipo | HMW adipo | 0,2176 |
| WT | adipo | HMW adipo |  | T-/- | adipo | without ligand | 0,0003 |
| WT | adipo | HMW adipo |  | WT | adipo | LDL | 0,0003 |
| WT | adipo | HMW adipo |  | T-/- | adipo | LDL | <0,0001 |
| T-/- | adipo | HMW adipo |  | T-/- | adipo | without ligand | 0,0052 |
| T-/- | adipo | HMW adipo |  | WT | adipo | LDL | 0,0068 |
| T-/- | adipo | HMW adipo |  | T-/- | adipo | LDL | <0,0001 |
| T-/- | adipo | without ligand |  | WT | adipo | LDL | 0,3930 |
| T-/- | adipo | without ligand |  | T-/- | adipo | LDL | <0,0001 |
| WT | adipo | LDL |  | T-/- | adipo | LDL | 0,0002 |
